# Trained immunity causes myeloid cell hypercoagulability

**DOI:** 10.1126/sciadv.ads0105

**Published:** 2025-03-07

**Authors:** Aisling M. Rehill, Seán McCluskey, Anna E. Ledwith, Tristram A. J. Ryan, Betül Ünlü, Gemma Leon, Hugo Charles-Messance, Edmund H. Gilbert, Paula Klavina, Emily A. Day, Judith Coppinger, Jamie M. O’Sullivan, Corrina McMahon, James S. O’Donnell, Annie M. Curtis, Luke A. J. O’Neill, Frederick J. Sheedy, Roger J. S. Preston

**Affiliations:** ^1^ Irish Centre for Vascular Biology, School of Pharmacy and Biomolecular Sciences, RCSI University of Medicine and Health Sciences, Dublin, Ireland.; ^2^ National Children’s Research Centre, Our Lady’s Children’s Hospital Crumlin, Dublin, Ireland.; ^3^ School of Biochemistry and Immunology, Trinity College Dublin, Dublin, Ireland.; ^4^ FutureNeuro SFI Research Centre, Royal College of Surgeons in Ireland, Dublin, Ireland.

## Abstract

The pathogenic basis for increased thrombotic risk in individuals with inflammatory diseases is poorly understood. Myeloid cell “trained immunity” describes persistent innate immune cell memory arising from prior exposure to an inflammatory stimulus, leading to an enhanced immune response to subsequent unrelated stimuli. We identify enhanced myeloid cell prothrombotic activity as a maladaptive consequence of trained immunity. Lipopolysaccharide (LPS) stimulation of macrophages trained previously with β-glucan or heme exhibited significantly enhanced procoagulant activity compared to macrophages stimulated with LPS alone, which was mediated by enhanced acid sphingomyelinase–mediated tissue factor decryption. Furthermore, splenic monocytes isolated from β-glucan–trained mice revealed enhanced procoagulant activity up to 4 weeks after β-glucan administration compared to monocytes from control mice over the same time period. Moreover, hematopoietic progenitor cells and bone marrow interstitial fluid from β-glucan–trained mice had enhanced procoagulant activity compared to control mice. Trained immunity and associated metabolic perturbations may therefore represent an opportunity for targeted intervention in immunothrombotic disease development.

## Teaser

Enhanced myeloid cell hypercoagulability is a maladaptive consequence of trained immunity.

## INTRODUCTION

The risk of venous thromboembolism is substantially elevated in patients with inflammatory disorders and hematological malignancies, which are often characterized by extended periods of chronic inflammation. These conditions, which include sickle cell disease (SCD) (*1*), inflammatory bowel disease (*2*), and myeloproliferative disorders (*3*), among others, have a high worldwide prevalence and substantial individual burden. Despite the elevated risk of thrombosis in affected individuals, the mechanistic basis for how chronic inflammatory disease might also promote hemostatic dysregulation in the long term is not understood. Elucidating the pathophysiological mechanisms that underlie increased thrombotic risk in individuals with inflammatory diseases is crucial to the development of new treatment options and prevention strategies.

Aberrant activation of peripheral and tissue-resident innate immune cells is strongly associated with the development of immunothrombosis (*4*, *5*). In addition to their acute response to pathogens, innate immune cells can retain a “memory” of past exposure to pathogens or inflammatory stimuli, predisposing these cells to a heightened pro-inflammatory response upon re-exposure to a nonspecific inflammatory event. This process is termed “trained immunity” or “innate immune memory” (*6*). Unlike an adaptive immune response, this phenomenon is not antigen specific and has likely evolved to enable innate immune cells to mount a robust response to subsequent pathogenic exposure. Well-characterized mediators of trained immunity include the Bacillus Calmette-Guerin (BCG) vaccine (*7*), *Candida albicans* (*8*), and β-glucan (*9*), a polysaccharide cell wall component. Moreover, sterile inflammation, caused by oxidized low-density lipoprotein (*10*) or free heme (*11*), can also confer innate immune memory.

To imprint innate immune memory, myeloid cells undergo extensive functional reprogramming via epigenetic and metabolic changes (*12*). β-Glucan–trained macrophages exhibit enhanced H3 histones trimethylated at Lys^4^ (H3K4me3) and H3 histones acetylated at Lys^27^ (H3K27ac) at the promoter and/or enhancer regions of key pro-inflammatory and metabolic genes (*9*, *13*, *14*). Profound changes in myeloid cell metabolism, including heightened glycolytic pathway activity, also accompany the phenotypic changes that arise from innate immune memory (*14*, *15*). In vivo, innate immune memory is mediated by phenotypic reprogramming of hematopoietic stem and progenitor cells (HSPCs). HSPC populations in the bone marrow (BM) rapidly proliferate in response to systemic inflammation or severe infection response (*8*, *9*, *16*). The induction of trained immunity promotes the expansion of HSPCs with a bias toward myelopoiesis, and the myeloid cells generated exhibit increased pro-inflammatory activity upon restimulation (*17*, *18*).

Although trained immunity is likely beneficial in boosting innate immune responses by generating heightened pro-inflammatory responses to subsequent pathogen exposure, maladaptive induction of trained immunity has also been linked to an increased propensity to develop and exacerbate chronic inflammatory disease. For example, metabolic dysfunction arising from a high-fat diet leads to HSPC myelopoietic bias and heightened myeloid cell pro-inflammatory activity in mice (*19*). Notably, maladaptive BM-trained immunity induced by periodontitis in mice exacerbates subsequent disease severity and pro-inflammatory activity when the same mice experience inflammatory arthritis, and vice versa (*20*). These data indicate that unrelated inflammatory disease activity can predispose to, or exacerbate, the development and severity of an unrelated inflammatory disease via long-term HSPC reprogramming and generation of myeloid cells with enhanced pro-inflammatory preparedness.

We still know little about how chronic or recurring inflammation shapes thrombotic risk in the long term. We hypothesized that this phenomenon may arise from individual inflammatory events that reprogram HSPC generation, leading to the synthesis of myeloid cells with a higher capacity for immunothrombotic activity. We demonstrate that exposure of monocytes and macrophages to mediators of trained immunity causes epigenetic and metabolic reprogramming, accelerating acid sphingomyelinase (ASMase)–mediated tissue factor decryption and enhancing myeloid cell antifibrinolytic activity. Moreover, we demonstrate that the induction of innate immune memory in mice causes BM reprogramming, which promotes myeloid cell and plasma procoagulant activity for weeks after the original training event.

## RESULTS

### β-Glucan–mediated training immunity results in enhanced macrophage procoagulant gene expression

**Fig. 1.**
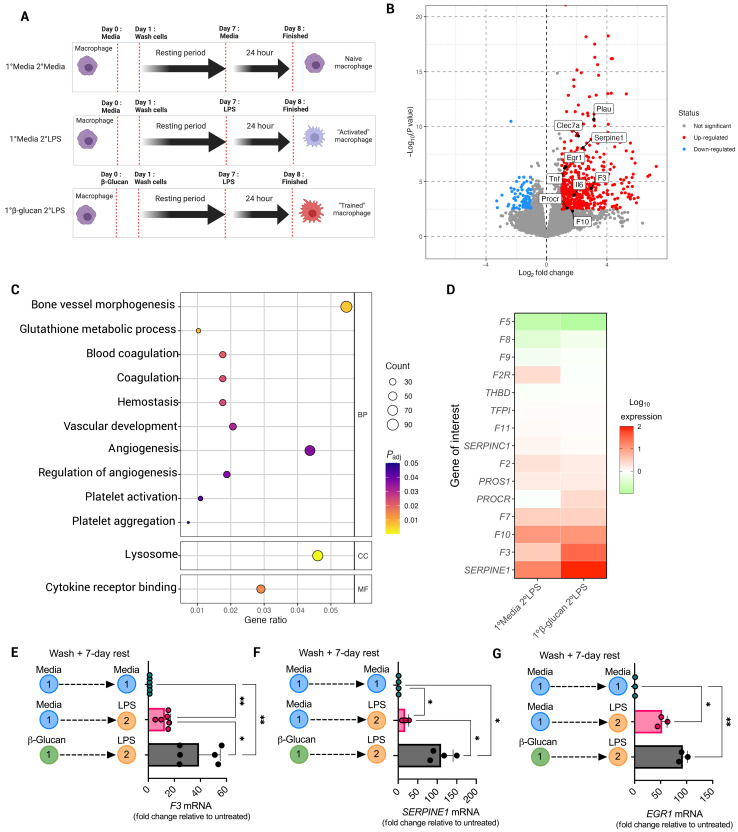
β-Glucan–induced trained immunity up-regulates coagulation-associated gene expression. (**A**) Schematic diagram outlining the protocol for inducing trained immunity in BMDMs. Briefly, BMDMs were pretreated with media or whole β-glucan particles (100 μg/ml) and left for 24 hours before cells were washed three times with PBS and left to rest for 1 week. On day 7, cells were restimulated with LPS (100 ng/ml) for 6 hours before RNA isolation. (**B**) Bulk RNA sequencing was performed on LPS-treated (*n* = 3) and β-glucan–trained BMDMs restimulated with LPS (*n* = 3), and differential gene expression analysis was performed between the groups. (**C**) Gene Ontology enrichment analysis of the gene sets significantly up-regulated in LPS-restimulated β-glucan–trained BMDMs compared to LPS-treated BMDMs. BP, biological processes; CC, cellular component; MF, molecular functions. (**D**) Heatmap showing coagulation-associated gene expression profiles for LPS-treated (1°Media 2°LPS) and LPS-restimulated β-glucan–trained (1°β-glucan 2°LPS) BMDMs following RT-qPCR analysis. Data were normalized for *RPS18* mRNA levels, and results were expressed as fold change compared to untreated BMDMs (1°Media 2°Media). RT-qPCR results for (**E**) *F3*, (**F**) *Serpine1*, and (**G**) *EGR1* mRNA levels. A one-way ANOVA was used to determine statistical significance with *P ≤ 0.05 and **P ≤ 0.01 for six independent experiments measured in duplicate.

In keeping with previous reports, β-glucan–treated BM-derived macrophages (BMDMs) released significantly more tumor necrosis factor–α (TNFα) upon secondary lipopolysaccharide (LPS) stimulation than when treated with LPS alone and exhibited increased glycolytic activity (fig. S1, A to C). To establish whether gene expression of coagulation-associated genes was altered in macrophages by prior induction of innate immune memory, we performed bulk RNA sequencing analysis using naïve and LPS-treated BMDMs that were previously exposed to either β-glucan or cell culture media 1 week prior (Fig. 1A). Differential expression of genes analysis of β-glucan–trained BMDMs showed significantly increased expression of the *TNF* and *IL6* genes [encoding TNFα and interleukin-6 (IL-6) cytokines] than untrained LPS-treated BMDMs, in keeping with previous studies (*14*, *21*) (Fig. 1B, fig. S2, and tables S2 and S3). We noted that β-glucan training also increased the expression of several coagulation-associated genes, including *F3*, *Serpine1*, and *Plau*, which encode tissue factor (TF), plasminogen activator inhibitor 1 (PAI-1), and urokinase plasminogen activator, respectively (Fig. 1, B and D, and table S2). Gene Ontology enrichment analysis of LPS-treated BMDMs that were previously treated with β-glucan demonstrated significantly up-regulated expression of gene sets associated with “coagulation” and “hemostasis” compared to BMDMs stimulated with LPS alone (Fig. 1C and fig. S2B). Further quantitative polymerase chain reaction (qPCR) analysis of restimulated β-glucan–trained BMDMs demonstrated that *F3* (Fig. 1E) and *Serpine1* (Fig. 1F) gene expression was significantly increased compared to LPS-treated BMDMs such that, relative to naïve BMDMs, *F3* expression in trained BMDMs was increased 31-fold compared to only a 3.5-fold increase in LPS-treated BMDMs (Fig. 1E). Moreover, β-glucan–mediated dectin-1 signaling induces macrophage expression of early growth response (EGR) transcription factors 1 to 3 (*22*), which act as upstream transcriptional regulators of LPS-induced myeloid cell *F3* and *Serpine1* expression (*23*). We found that β-glucan training before LPS stimulation caused significantly increased *EGR1* expression compared to LPS-stimulated BMDMs (Fig. 1, B and G).

### β-Glucan–mediated training results in myeloid cell hypercoagulability

**Fig. 2.**
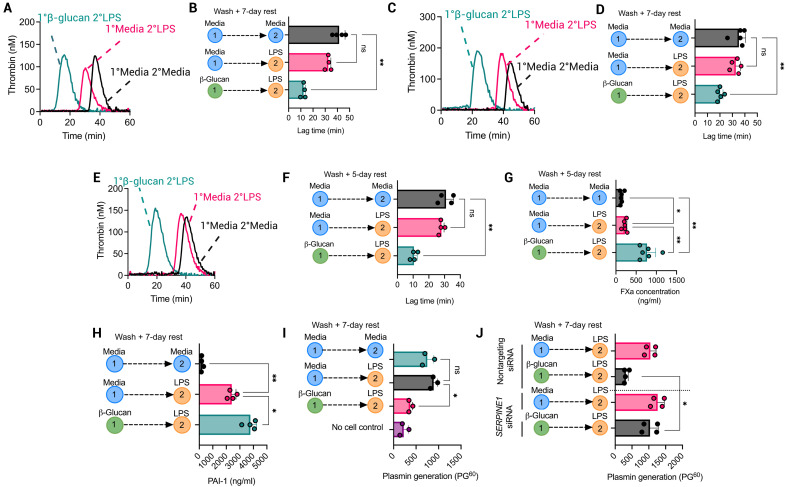
β-Glucan–induced trained immunity enhances myeloid cell procoagulant activity. Cells were pretreated with media or whole β-glucan particles (100 μg/ml) and left for 24 hours before cells were washed three times with PBS and left to rest for 1 week. On day 7, cells were restimulated with LPS (100 ng/ml) for 24 hours. A cell-based TGA was performed with cells as the sole source of TF activity in 80 μl of FXII-deficient plasma, 20 μl of MP reagent, and 20 μl of thrombin substrate added to initiate the reaction. (**A**) Thrombogram for TGA performed with BMDMs from each group and (**B**) associated lag times calculated. ns, not significant. (**C**) Thrombogram for TGA performed in the presence of BMDM supernatants (80 μl) and (**D**) associated lag times. (**E**) Thrombogram for TGA performed with β-glucan–trained primary human monocytes and (**F**) associated lag times. (**G**) A FXa generation assay was performed in the presence of primary human monocytes incubated with FVIIa (0.5 μg/ml) and FX (10 μg/ml) for 30 min. An FXa substrate was added to detect FXa generated as a consequence of TF activity. (**H**) PAI-1 protein levels measured by ELISA in β-glucan–trained BMDMs. (**I**) tPA-mediated plasmin generation in the presence of β-glucan–trained BMDMs was measured using a plasmin-specific fluorogenic substrate, and the fluorescence reading after 60 min (PG^60^) was determined. (**J**) BMDMs were transfected with *Serpine1* siRNA or nontargeting siRNA (both 20 nM) before β-glucan training and LPS treatment. tPA-mediated plasmin generation in the presence of these cells was then assessed. A paired *t* test or one-way ANOVA was used where appropriate to determine statistical significance with *P ≤ 0.05 and **P ≤ 0.01 for three to six independent experiments measured in duplicate.

On the basis of these findings, we sought to determine the impact of β-glucan–mediated innate immune memory on macrophage procoagulant activity in plasma. Evaluation of β-glucan–trained BMDMs via a modified cell-based thrombin generation assay (TGA) (*24*) revealed that LPS-treated BMDMs previously trained with β-glucan had significantly increased procoagulant activity compared to LPS-treated BMDMs (Fig. 2A), causing a 29-min shortening of lag time compared to naïve BMDMs and a 22-min reduction in lag time compared to LPS-treated BMDMs (Fig. 2B). Notably, no significant change in procoagulant activity was observed in BMDMs 24 hours after β-glucan exposure alone (fig. S3A), and *F3* gene expression was only minimally increased (fig. S3B). This β-glucan–mediated increased procoagulant activity was blunted by the presence of an anti-dectin-1 antibody before β-glucan stimulation (fig. S3, E and F) or Toll-like receptor 4 (TLR4) inhibition before LPS restimulation (fig. S3, G and H). In addition, increased procoagulant activity persisted in β-glucan–trained BMDMs when the rest period was extended to 2 weeks (fig. S3, I and J). To confirm that the enhanced procoagulant response in β-glucan–trained BMDMs was not elicited uniquely by LPS restimulation, we restimulated β-glucan–trained BMDMs with the TLR1/2 agonist PAM3CSK4. A similar increase in *F3* gene expression (31-fold) and a decrease in lag time (29-min) were observed in PAM3CSK4-restimulated trained BMDMs to that observed with LPS-restimulated trained BMDMs (fig. S4, A to D).

Supernatants from each treatment group were collected and assessed. Notably, a 12-min shortening of lag time was observed in the presence of supernatants from restimulated β-glucan–trained BMDMs compared to supernatants from LPS-treated BMDMs, suggesting an increase in the presence of procoagulant extracellular vesicles (EVs) in supernatants from β-glucan–trained BMDMs (Fig. 2, C and D). Nanoparticle tracking analysis confirmed an increased concentration of EVs from β-glucan–trained BMDM supernatants compared to untreated supernatants (fig. S5, A and B). Western blot analysis showed that both CD9, an EV marker, and TF were present in the supernatants of treated BMDMs (fig. S5C). These data indicate that β-glucan–induced innate immune memory increases LPS-induced TF procoagulant activity in macrophages and promotes procoagulant TF-containing EV release.

Similarly, restimulated β-glucan–trained purified human monocytes shortened the lag time to thrombin generation by 20 min compared to naïve monocytes alone (Fig. 2, E and F). Factor Xa (FXa) generation was also significantly enhanced in the presence of β-glucan–trained human monocytes compared to untreated or LPS-treated monocytes (Fig. 2G). FXa generation was negligible in the absence of FVIIa and significantly decreased in the presence of a TF neutralizing antibody, confirming that the observed effect was TF dependent (fig. S6, A and B). Recent studies have implicated T lymphocytes in enabling trained immunity in human monocytes (*25*). β-Glucan–induced hypercoagulability was observed in both column-purified CD14^+^ monocytes and adherent PBMCs (fig. S5, C to G), suggesting that in contrast to their role in trained immunity, the presence of interferon-γ–producing lymphocytes is not required for enhanced thrombin generation and procoagulant activity in β-glucan–trained monocytes.

### β-Glucan–mediated training enhances LPS-mediated impairment of myeloid cell fibrinolytic activity

As restimulated β-glucan–trained BMDMs up-regulated *Serpine1* expression and doubled PAI-1 released compared to LPS-treated BMDMs (Figs. 1F and 2H), we assessed the impact of restimulated β-glucan–trained BMDMs on macrophage regulation of fibrinolytic activity via altered plasmin generation. LPS restimulation of β-glucan–trained BMDMs exhibited significantly enhanced inhibition of plasmin generation compared to LPS-treated BMDMs (Fig. 2I). Subsequent *Serpine1* inhibition by small interfering RNA (siRNA) knockdown confirmed inhibition of plasmin generation that resulted from increased PAI-1 production (Fig. 2J). *Serpine1* gene expression was unchanged after 24 hours of β-glucan exposure and did not affect plasmin generation alone (fig. S3, C and D). These data indicate that restimulated β-glucan–trained BMDMs have a significantly higher procoagulant and antifibrinolytic activity than when directly stimulated by LPS alone.

### The protoporphyrin IX ring in free heme mediates trained hypercoagulability

We next sought to determine whether trained hypercoagulability could be induced by endogenous damage-associated molecular pattern mediators of trained immunity. Free heme has recently been shown to impart innate immune memory in myeloid cells (*11*). Free heme treatment followed by LPS restimulation 7 days later caused a significant increase in TNFα production and metabolic reprogramming in both human monocytes and murine BMDMs, as previously reported (*11*) (fig. S6, A, B, and E). To first investigate the structural component(s) of free heme responsible for this response, we coincubated individual heme structural components, protoporphyrin IX (PPIX) and iron (ferric nitrilotriacetate; Fe-NTA), with BMDMs, washed them out, and restimulated them with LPS 7 days later. PPIX pretreatment caused a similar increase in TNFα production to that of free heme following LPS restimulation (fig. S7C), whereas Fe-NTA–pretreated BMDMs did not increase TNFα production after LPS restimulation (fig. S7D). Protoporphyrin-trained BMDMs also exhibited increased basal glycolysis compared to LPS-treated BMDMs, whereas Fe-NTA–treated BMDMs did not cause any change in basal glycolysis (fig. S7e). These results demonstrate that the protoporphyrin ring of heme induces metabolic reprogramming characteristic of trained myeloid cells.

**Fig. 3.**
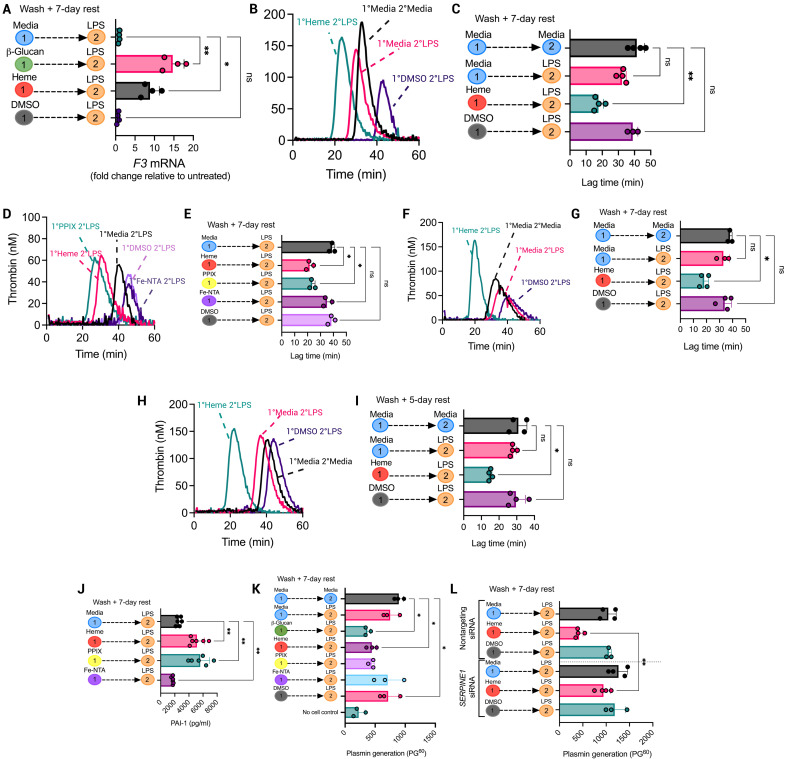
Free heme induces trained hypercoagulability in myeloid cells via the protoporphyrin IX (PPIX) ring. BMDMs and human monocytes were trained with 50 μM heme, 50 μM PPIX, 50 μM Fe-NTA, or DMSO (dimethyl sulfoxide) vehicle control. After 24 hours, cells were washed and rested for 7 days. Cells were then restimulated with LPS (100 ng/ml) for 6 hours for mRNA analysis and 24 hours for protein analysis and functional assays. (**A**) *F3* mRNA levels in heme-trained BMDMs were determined by RT-qPCR. Data were normalized to *RPS18* mRNA levels, and results were expressed as fold change relative to untreated BMDMs (1°Media 2°Media). (**B**) Thrombogram for TGA performed in the presence of heme-trained BMDMs and (**C**) associated lag times. (**D**) Thrombogram for TGA performed in the presence of PPIX- or Fe-NTA–trained BMDMs and (**E**) associated lag times. (**F**) Thrombogram for TGA performed in the presence of heme-trained BMDM supernatants and (**G**) associated lag times. (**H**) Thrombogram for TGA performed in the presence of heme-trained human monocytes and (**I**) associated lag times. (**J**) PAI-1 levels were measured by ELISA in heme-, PPIX-, and Fe-NTA–trained BMDMs. (**K**) tPA-mediated plasmin generation in the presence of heme-, PPIX-, and Fe-NTA–trained BMDMs was measured using a plasmin-specific fluorogenic substrate, and the fluorescence reading after 60 min (PG^60^) was determined. (**L**) BMDMs were transfected with *Serpine1* siRNA or nontargeting siRNA (both 20 nM) before heme training and LPS treatment. tPA-mediated plasmin generation in the presence of treated BMDMs was then performed. A paired *t* test or one-way ANOVA was used where appropriate to determine statistical significance with *P ≤ 0.05 and **P ≤ 0.01 for three to six independent experiments measured in duplicate.

*F3* gene expression in restimulated BMDMs exposed to heme 1 week prior was increased ninefold relative to LPS-treated BMDMs (Fig. 3A). Heme-trained BMDMs subsequently treated with LPS induced significantly shorter lag times to thrombin generation than BMDMs treated with LPS alone (Fig. 3, B and C). This effect largely depended on PPIX, which shortened the lag time by 16 min compared to when naïve BMDMs were present (Fig. 3, D and E). Also, supernatants from restimulated heme-trained BMDMs caused a 15-min shortening of lag time compared to LPS-treated BMDMs (Fig. 3, F and G), suggesting that heme-induced innate immune memory causes the enhanced release of procoagulant EVs upon re-exposure to pro-inflammatory stimuli. Similarly, heme pretreatment induced a higher procoagulant activity in the presence of purified human monocytes, resulting in a 13-min shortening in lag time compared to LPS-treated BMDMs (Fig. 3, H and I). Although heme has previously been reported to signal via TLR4 (*26*), TLR4 neutralization had no impact on heme training–induced procoagulant activity in BMDMs (fig. S7, F and G).

As with β-glucan–trained BMDMs, PAI-1 production was significantly increased in heme-trained BMDMs compared to LPS-treated BMDMs, which was similarly dependent on the protoporphyrin ring (Fig. 3J). Both heme- and protoporphyrin-trained BMDMs reduced plasmin generation by approximately half of that generated by LPS-treated BMDMs, but there was no change in plasmin generation activity in Fe-NTA–treated BMDMs (Fig. 3K). siRNA-mediated knockdown of *Serpine1* in heme-trained BMDMs resulted in recovery of plasmin generation comparable to LPS-treated BMDMs, confirming PAI-1–dependent inhibition of plasmin generation in restimulated heme-trained BMDMs (Fig. 3L). These results demonstrate that trained hypercoagulability is a common feature of myeloid cell innate immune cell memory.

### Epigenetic and metabolic macrophage reprogramming is essential for trained hypercoagulability

**Fig. 4.**
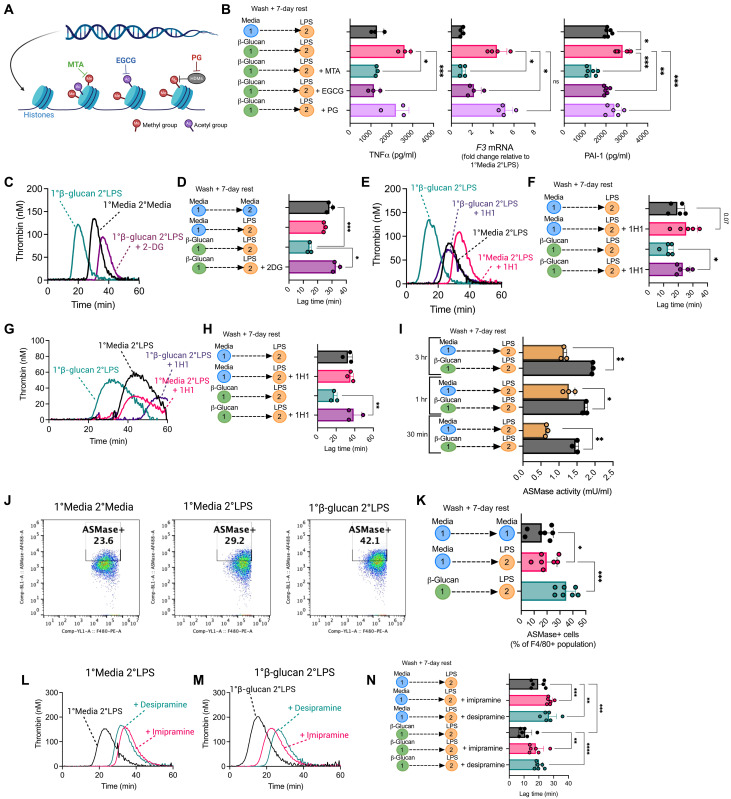
β-Glucan–induced trained hypercoagulability requires epigenetic and metabolic reprogramming. (**A**) Schematic diagram illustrating chemical epigenetic inhibitor targets. (**B**) BMDMs were incubated with 1 mM MTA, 100 μM EGCG, or 12 mM PG for 1 hour before training with β-glucan (100 μg/ml) for 24 hours. Cells were washed three times, and growth media were supplemented with epigenetic inhibitors added for the rest period (100 μM MTA, 100 μM EGCG, and 12 mM PG). On day 7, cells were restimulated with LPS (100 ng/ml). *TNFA* and *F3* mRNA levels were determined by RT-qPCR, and PAI-1 levels were determined by ELISA. (**C**) BMDMs were pretreated for 3 hours with 10 mM 2-DG before the β-glucan training protocol. After LPS restimulation on day 7, cells were assessed in a TGA to generate a thrombogram and (**D**) lag times. (**E** and **F**) BMDMs or (**G** and **H**) BMDM supernatants were incubated with anti-TF antibody (1H1) for 1 hour before TGA analysis and lag-time measurement. (**I**) ASMase activity in trained BMDMs was measured by fluorogenic ASMase activity assay. Trained cells were restimulated with LPS for 30 min, 1 hour, or 3 hours before cell lysis. (**J** and **K**) ASMase cell surface expression on BMDMs was determined by flow cytometry in F4/80^+^ BMDM populations. (**L**) β-Glucan–trained and (**M**) untreated BMDMs were incubated with 10 μM desipramine or imipramine for 1 hour before LPS restimulation on day 7. TGA analysis was then performed, and the (**N**) lag time was determined. A paired *t* test or one-way ANOVA was used where appropriate to determine statistical significance with *P ≤ 0.05, **P ≤ 0.01, and ***P ≤ 0.001 for three to seven independent experiments measured in duplicate.

Given the role of transcriptomic, epigenomic, and metabolic reprogramming in enabling innate immune memory, we tested whether similar adaptations would be necessary for trained hypercoagulability. First, we used inhibitors that target histone methylation and acetylation (Fig. 4A). In common with previous reports, the histone methyltransferase inhibitor, methyl thioadenosine (MTA), and the histone acetyltransferase inhibitor, epigallocatechin-3-gallate (EGCG), reduced macrophage TNFα release from restimulated β-glucan–trained BMDMs, whereas the histone demethylase inhibitor, paragyline (PG), had no significant impact, in keeping with a prior report (*27*) (Fig. 4B). Furthermore, *F3* expression was reduced five- and twofold with MTA and EGCG pretreatment in LPS-restimulated, β-glucan–trained BMDMs (Fig. 4B). PAI-1 production was also significantly impaired in MTA-pretreated trained BMDMs (Fig. 4B). Epigenetic inhibition in LPS-treated macrophages had a minimal effect on TNFα, PAI1, and *F3* expression (fig. S8). These results suggest that posttranslational histone modifications arising from β-glucan–induced innate immune memory are required for trained hypercoagulability and suggest a prominent role for histone methylation in the enhanced procoagulant activity of β-glucan–trained BMDMs. Innate immune memory also causes extensive rewiring of intracellular metabolic pathways, leading to enhanced macrophage glycolysis (fig. S1C). To examine the role of glycolysis in trained hypercoagulability, we used 2-deoxy-glucose (2-DG), a glucose analog that acts as a competitive inhibitor of hexokinase. 2-DG treatment of β-glucan–trained BMDMs significantly prolonged thrombin generation, restoring lag time to that observed in the presence of untreated BMDMs (Fig. 4, C and D), demonstrating that β-glucan–induced enhancement of macrophage glycolytic activity is necessary for trained hypercoagulability.

### ASMase procoagulant activity is up-regulated in β-glucan–trained BMDMs

We next sought to investigate the mechanisms responsible for increased procoagulant activity observed in restimulated β-glucan–trained macrophages. The role of TF activity in mediating the enhanced procoagulant activity of restimulated β-glucan–trained BMDMs was confirmed using an anti-TF monoclonal antibody, which extended lag time to that of naïve BMDMs in cell (Fig. 4, E and F)– and supernatant (Fig. 4, G and H)–triggered thrombin generation. TF surface expression was increased in all treatment groups following LPS restimulation; however, despite the significant difference in procoagulant activity, there was unexpectedly little difference in BMDM TF surface expression between LPS-treated BMDMs and LPS-treated BMDMs that had been previously exposed to β-glucan (fig. S9A). TF typically exists in an encrypted, noncoagulant state, and additional mechanisms are required to decrypt TF into a procoagulant conformation. Phosphatidylserine (PS) externalization is important for TF procoagulant activity and coagulation factor complex assembly necessary for thrombin generation and clot formation (*28*, *29*). PS surface expression was investigated to examine whether PS externalization was enhanced in β-glucan–trained BMDMs. LPS restimulation increased PS surface expression in all treatment groups; however, no significant difference was observed between trained BMDMs and LPS-treated cells (fig. S9, B and C). Furthermore, a cell-based prothrombinase activity assay showed no significant difference in capacity to support prothrombinase activity between LPS-treated and β-glucan–trained BMDMs (fig. S9D). Furthermore, changes in TF disulfide bond formation did not contribute to enhanced procoagulant activity in trained BMDMs, as treatment of macrophages with the protein disulfide isomerase inhibitor rutin did not affect β-glucan training–mediated accelerated thrombin generation (fig. S9, E to G). We next investigated how induction of innate immune memory may affect ASMase activity, which enhances TF procoagulant activity via TF decryption in macrophages (*30*). Notably, β-glucan–trained BMDMs subsequently exposed to LPS had significantly elevated ASMase activity compared to BMDMs treated with LPS alone (Fig. 4I). ASMase cell surface expression was also significantly increased on β-glucan–trained BMDMs compared to LPS-treated BMDMs (Fig. 4, J and K). In addition, preincubation with the ASMase inhibitor desipramine or imipramine before LPS restimulation significantly prolonged lag time on BMDMs (Fig. 4, L to N). These results suggest that the enhanced TF activity observed in restimulated β-glucan–trained macrophages derives from enhanced TF decryption mediated by a training-mediated enhancement of cell surface ASMase activity.

### Induction of central trained immunity induces the generation of hypercoagulable myeloid cells

**Fig. 5.**
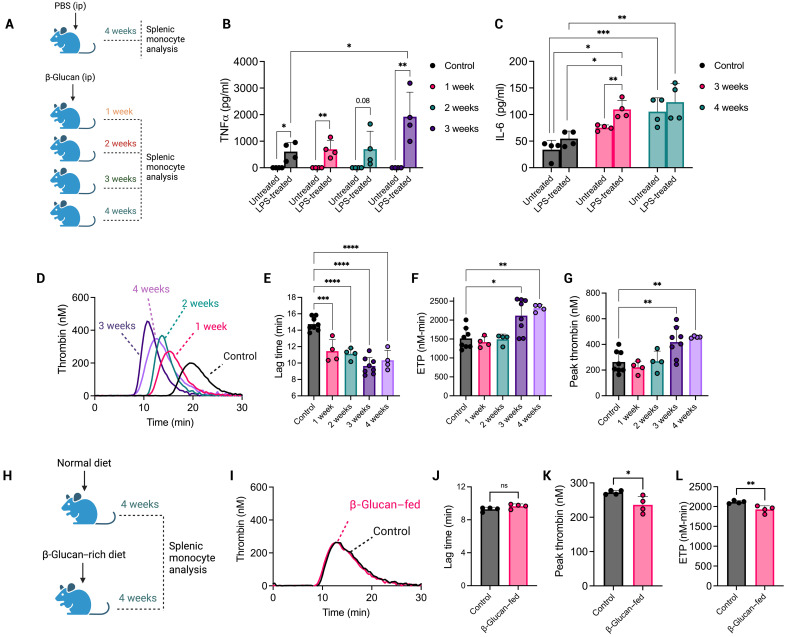
Splenic monocytes from β-glucan–administered mice display enhanced procoagulant activity weeks after administration. (**A**) Schematic depiction of the experimental outline to invoke trained immunity in vivo. Briefly, mice were injected with either PBS or whole β-glucan particles 1 to 4 weeks before sacrifice. CD115^+^ splenic monocyte population was then isolated. Splenic monocytes were left untreated or restimulated with LPS (100 ng/ml) ex vivo for 24 hours. ip, intraperitoneally. (**B**) TNFα and (**C**) IL-6 levels released from splenic monocytes were determined by ELISA. (**D**) A TGA was performed in the presence of splenic monocytes isolated from mice administered β-glucan 1 to 4 weeks previously. (**E**) Lag time, (**F**) ETP, and (**G**) peak thrombin were also determined. (**H**) In parallel, mice were fed a standard diet or a diet supplemented with dietary β-glucan. Splenic monocytes were then isolated and assessed by TGA to give (**I**) a thrombogram, (**J**) lag time, (**K**) peak thrombin, and (**L**) ETP. A paired *t* test or one-way ANOVA was used where appropriate to determine statistical significance with *P ≤ 0.05, **P ≤ 0.01, ***P ≤ 0.001, and ***P ≤ 0.0001 for four to eight mice with samples measured in duplicate.

Systemic β-glucan training causes long-lasting epigenetic and metabolic alterations to hematopoietic progenitor cells in the BM that favor myelopoiesis (*17*). To establish whether induction of central trained immunity would result in the generation of mature myeloid progeny with an increased capacity for procoagulant activity, mice were administered whole β-glucan particles by intraperitoneal injection and left for between 1 and 4 weeks (Fig. 5A). Splenic monocytes were then isolated from mice 1 to 4 weeks after β-glucan administration. To confirm that the isolated splenic monocytes exhibited characteristic features of trained immunity, TNFα production was measured before and after LPS restimulation. TNFα was not detected in monocytes before LPS restimulation; however, there was a significant increase in TNFα production in LPS restimulated monocytes from β-glucan–treated mice, with the most significant increase occurring in monocytes derived from mice that had received β-glucan 3 to 4 weeks prior (Fig. 5B). IL-6 was also significantly increased in β-glucan–treated mice both before and after ex vivo LPS treatment in mice that had received β-glucan 3 to 4 weeks prior (Fig. 5C). Next, we compared the TF-mediated procoagulant activity of splenic monocytes from either saline- or β-glucan–administered mice. Splenic monocytes from mice treated with β-glucan 3 to 4 weeks previously exhibited a ~6-min shorter lag time than monocytes from saline-administered mice. This phenomenon was observed in the presence and absence of ex vivo LPS restimulation (Fig. 5, D and E, and fig. S10). Peak thrombin and endogenous thrombin potential (ETP) were also increased in the presence of monocytes isolated from mice treated with β-glucan 3 or 4 weeks previously compared to saline-administered mice, suggesting that central trained immunity in vivo causes the generation of monocytes with increased capacity for thrombin generation (Fig. 5, F and G). This procoagulant effect plateaued at 3 weeks, with no further enhancement in TGA parameters after 4 weeks (Fig. 5, D to G). The longer the interval between β-glucan administration and animal sacrifice, the more significant the increase in splenic monocyte procoagulant activity was observed. We also assessed whether dietary β-glucan had the same impact on splenic monocyte procoagulant activity (Fig. 5H) but found no difference in splenic monocyte–mediated thrombin generation parameters between β-glucan–fed mice and control diet–fed mice (Fig. 5, I to L).

### Central trained immunity results in a hypercoagulable BM environment and increased plasma procoagulant activity

**Fig. 6.**
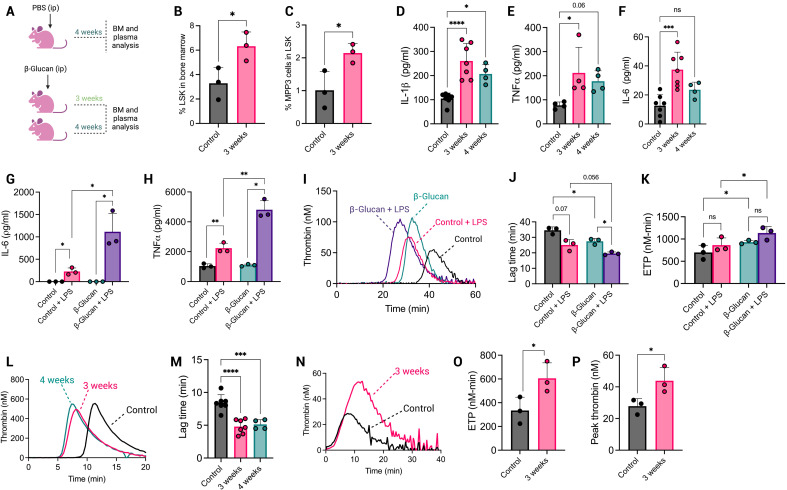
β-Glucan–induced central trained immunity results in a BM environment with enhanced procoagulant activity. (**A**) Briefly, mice were injected with either PBS or whole β-glucan particles 1 to 4 weeks before sacrifice. Plasma was collected via the retro-orbital sinus. BM interstitial fluid and HSCs were harvested. BM HSPC populations were analyzed by flow cytometry for (**B**) % LSK^+^ cells in BM and (**C**) % MPP3 myeloid-biased progenitor cells in the LSK^+^ compartment. (**D**) IL-1β, (**E**) TNFα, and (**F**) IL-6 cytokine levels were determined in BM interstitial fluid by ELISA. (**G**) IL-6 and (**H**) TNFα levels released from cultured BMDMs without or with ex vivo LPS restimulation were determined by ELISA. Cultured BMDMs without or with ex vivo LPS restimulation were included in a TGA to generate (**I**) a thrombogram, (**J**) lag time, and (**K**) ETP values. (**L**) TGA was performed in the presence of BM interstitial fluid to give a thrombogram and (**M**) associated lag times. (**N**) TGA analysis was performed with mouse platelet-poor plasma isolated from trained and control mice. Each well contained 20 μl of plasma, 20 μl of TBS, and 20 μl of FluCa thrombin substrate, and TGA parameters (**O**) ETP and (**P**) peak thrombin values are shown. A paired *t* test or one-way ANOVA was used where appropriate to determine statistical significance with *P ≤ 0.05, **P ≤ 0.01, ***P ≤ 0.001, and ***P ≤ 0.0001 for four to eight mice with samples measured in duplicate.

The detection of hypercoagulable myeloid cells that had been synthesized weeks after β-glucan administration suggested the development of a BM environment that favored the generation of procoagulant mature myeloid cells. We next evaluated mouse BM progenitor cell populations in saline-treated mice and mice administered β-glucan 3 weeks before sacrifice (Fig. 6A). As previously reported (*17*), there were a significantly increased percentage of hematopoietic progenitor cells (LSK; Lin^−^cKit^+^Sca1^+^) in the BM of β-glucan–administered mice compared to saline-treated mice (Fig. 6B) and, within this population, a significant increase in myeloid-biased multipotent progenitor 3 (MPP3; Flt3^−^CD48^+^CD150^−^LSK) cells (Fig. 6C; see fig. S11 for the gating strategy). Notably, there was no significant increase in lymphoid-biased progenitors (fig. S12, A to D). As IL-1β enhances hematopoietic activity induced by trained immunity (*9*), we measured IL-1β in the BM interstitial fluid of β-glucan–administered and saline-administered control mice. IL-1β was significantly increased in BM interstitial fluid isolated from β-glucan–administered mice (Fig. 6D), with a similar pattern observed for TNFα and IL-6 levels (Fig. 6, E and F). Cytokine production from BMDMs isolated and cultured from β-glucan– and saline-administered mouse BM was also measured in the presence or absence of ex vivo LPS restimulation. TNF and IL-6 release was significantly increased from BMDMs isolated from β-glucan–administered mice upon LPS restimulation compared to BMDMs from saline-administered control mice (Fig. 6, G and H). BMDMs generated from β-glucan–administered mice also supported enhanced thrombin generation compared to saline-treated mice both with and without ex vivo LPS treatment, as seen via reduced lag time and enhanced ETP (Fig. 6, I to K).

Given the increased pro-inflammatory cytokine production in β-glucan–administered mouse BM and the possibility of increased procoagulant EVs, we assessed the procoagulant activity of cell-free BM interstitial fluid from β-glucan–administered and saline-administered mice. Notably, BM interstitial fluid from β-glucan–administered mice was able to drive quicker clot formation, shortening the lag time by ~5 min compared to BM fluid from saline-administered mice, suggesting the increased presence of procoagulant EVs within the mouse BM (Fig. 6, L and M). Last, to assess whether prior β-glucan administration caused a systemic increase in plasma procoagulant activity, we assessed thrombin generation in platelet-poor plasma derived from β-glucan–administered and saline-treated control mice without the addition of an exogenous thrombin generation trigger. Plasma from mice administered β-glucan 3 weeks prior exhibited significantly higher peak thrombin and ETP than plasma from saline-administered control mice (Fig. 6, N to P); however, no significant change in thrombin-antithrombin complexes was observed in plasma or BM interstitial fluid (fig. S12, E and F). Collectively, these results indicate that central trained immunity causes the adoption of a procoagulant BM microenvironment that results in the generation of myeloid cells with a higher capacity for procoagulant activity and the release of procoagulant EVs within the BM and the periphery.

## DISCUSSION

Immune cell adaptations in response to infection or inflammation are critical for host survival. Adaptive immunity leads to the development of antigen-specific lymphocytes that lay primed to respond to subsequent similar immune challenges. Innate immune memory, however, enables peripheral myeloid cells to respond to nonspecific pathogens with enhanced vigor. This transformation is mediated by epigenetic and metabolic reprogramming that trains cells for subsequent immune responses to unrelated challenges. Although this feature remains beneficial in strengthening subsequent first-line defenses to infection and malignancy, the “shortening of the inflammatory fuse” has also been linked to the development of autoinflammatory diseases (*31*, *32*). In this study, we demonstrate the prothrombotic consequences of inducing trained immunity in myeloid cells. While crucial for enhanced trained immune response, these adaptations inadvertently increase the propensity of trained myeloid cells to promote aberrant thrombin generation via dysregulated TF expression and decryption, combined with inhibition of macrophage profibrinolytic activity. Moreover, induction of central trained immunity by β-glucan administration in mice causes the generation of a pro-inflammatory BM environment defined not only by increased pro-inflammatory cytokine production but also by HSPC reprogramming that leads to the generation of myeloid cells with increased capacity for thrombin generation and increased procoagulant activity in BM interstitial fluid and plasma.

TF activity in myeloid cells rapidly increases upon exposure to pathogen- or damage-associated molecular patterns, but its expression typically subsides after 24 hours (*33*, *34*). *F3* gene expression in activated myeloid cells is regulated by several transcription factors, including nuclear factor κB, activating protein 1 family members, and EGR1, that bind at the *F3* promoter and enhancer regions (*35*–*37*). Our initial transcriptomic studies in BMDMs identified significantly increased *EGR1* expression when BMDMs were stimulated with β-glucan 1 week before LPS exposure compared to BMDMs that were only exposed to LPS. In addition to regulating pro-inflammatory cytokine expression in response to infection or injury, EGR1 is considered a master regulator transcription factor for many pro-inflammatory genes, including genes encoding TF and PAI-1 (*23*, *38*). Pertinently, previous studies have shown that *C. albicans*–derived β-glucan stimulation of the dectin-1 receptor results in the activation of EGR1, EGR2, and EGR3 (*22*). Given that β-glucan–mediated trained immunity is mediated through dectin-1 signaling (*14*), EGR1 induction by β-glucan is likely central to subsequent activation of coagulation and antifibrinolytic gene programs in myeloid cells.

Durable innate immune memory in peripheral myeloid cells is made possible by the development of central trained immunity, in which a training agent increases BM IL-1β and granulocyte-macrophage colony-stimulating factor to promote myelopoiesis via HSPC changes in glucose metabolism and cholesterol biosynthesis pathways (*17*, *39*). In assessing whether the observed β-glucan–mediated higher capacity for procoagulant activity was a transient phenomenon or could be imparted long term, we observed that in mice, isolated splenic monocyte procoagulant activity increased with time after β-glucan administration up to 3 weeks, before plateauing at 4 weeks. Given that the lifespan of monocytes in the periphery is 1 to 7 days, the observed hypercoagulability of these monocytes is in keeping with the synthesis of new centrally trained peripheral myeloid cells with enhanced procoagulant, pro-inflammatory features. Isolated monocytes from β-glucan–administered mice required ex vivo LPS stimulation to prompt enhanced TNF secretion, whereas IL-6 expression from isolated monocytes increased with the duration of time since β-glucan administration, with only a limited increase in response to additional LPS. Similarly, TF-driven thrombin generation mediated by isolated monocytes from β-glucan–administered mice was only minimally enhanced by additional LPS. This suggests that β-glucan–induced central trained immunity, in addition to skewing BM activity toward myelopoiesis, also causes the generation of monocytes with a constitutively higher capacity for procoagulant activity. Although the duration of trained hypercoagulability is currently unknown, it is possible that it could persist for longer than the 3 to 4 weeks observed in our study. Previous studies have shown that the epigenetic and metabolic reprogramming associated with trained immunity in vivo typically persists for weeks to months (*19*, *40*, *41*); however, in human studies, particularly in relation to BCG vaccination–mediated trained immunity, hyperresponsive innate immune cells can persist for years (*42*). Some studies have also suggested that trained immunity may be inherited from prior generations. One study showed that the progeny of mice that survived an infection of *C. albicans* had enhanced survival when subjected to endotoxin challenge, and this protection was conferred via epigenetic and transcriptional changes in the myeloid progenitor cell BM cell populations (*43*). In addition, in humans, the beneficial effects of the BCG vaccine in newborns were enhanced when the mother had also received the BCG vaccine (*44*).

Similarly, we observed significantly increased procoagulant activity in BM interstitial fluid from mice administered β-glucan >3 weeks before sacrifice. This suggests the increased release of procoagulant microparticles into the BM microenvironment during central training. Although the cellular sources of procoagulant EVs in the BM have not yet been determined, previous studies have shown that hematopoietic stem cell (HSC) derived TF-containing EVs contribute to thrombus formation following laser-induced vessel wall injury (*45*). In addition, other studies have shown that depletion of HSC-derived TF protects against endotoxin challenge by reducing coagulation activation (*46*, *47*). Increased plasma procoagulant activity was also observed, although whether this is a consequence of TF-containing EVs generated in the BM entering the bloodstream, or whether TF-containing EVs were instead more readily released from newly synthesized monocytes recently generated in the BM or other BM-derived cells, is not yet known.

Trained hypercoagulability in myeloid cells was not limited to β-glucan training, as we also observed the same similar procoagulant and antifibrinolytic response in cells trained with free heme. Heme was recently shown to also induce myeloid cell–trained immunity in monocytes and macrophages. This is mediated by epigenetic reprogramming, specifically through H3K27ac, which was enriched at hundreds of genomic loci in heme-trained BMDMs (*11*). Increased circulating free heme via hemolysis arises in a number of inflammatory diseases but is particularly important in the context of SCD (*48*). Circulating immune cells from individuals with SCD are often characterized by a poorly understood heightened pro-inflammatory state, which contributes to vaso-occlusive crisis in affected individuals (*49*, *50*). Sickle red blood cells (RBCs) are prone to hemolysis, releasing RBC intracellular components into the intravascular space to activate the surrounding endothelium and circulating leukocytes (*51*). In particular, heme released from lysed RBCs can induce pro-inflammatory cytokine release from monocytes, further amplifying the pro-inflammatory conditions within the vasculature of patients with SCD, but the molecular basis for this is not well understood (*52*, *53*). Hemoglobin released from lysed RBCs contains a reactive ferric PPIX group, which can trigger vascular and organ dysfunction and cause adverse clinical effects, including renal failure, immune dysregulation, atherosclerosis, vascular injury, and endothelial dysfunction (*54*, *55*). Free heme has also been shown to contribute to TF-dependent thrombin generation in a mouse model of SCD (*56*). Despite this, hemopexin, which neutralizes free heme, only partially attenuated thrombin generation in sickle cell mice, suggesting the presence of additional contributory mechanisms (*56*). Our data suggest that increased free heme may also promote both peripheral and central trained immunity to further exacerbate the risk of coagulopathy in patients with SCD. These data could also provide mechanistic insights into the pathophysiology of other hemolytic conditions, such as thrombotic thrombocytopenic purpura and hemolytic uremic syndrome, which are characterized by increased labile heme in the circulation and an increased risk of thrombotic complications (*57*, *58*).

There are several limitations to our study. Our study uses a classical mediator of trained immunity, β-glucan, to induce myeloid cell training in vivo, and further studies are required to characterize whether other reported endogenous agents, such as oxidized low-density lipoprotein (*59*), aldosterone (*60*), or high-fat diet (*19*), might also impart increased myeloid cell hypercoagulability in mice. Similarly, our current studies provide a platform for assessment of whether inflammatory disease itself, rather than specific training agents, promotes a training-associated increased risk of deep vein thrombosis in appropriate model systems and, ultimately, patient cohorts. Furthermore, additional studies are required to determine whether innate immune training of other cell types associated with the propagation and regulation of blood coagulation (*61*–*63*) may also contribute to increased thrombotic risk.

Collectively, these findings raise important questions about the potential contribution of immune cell adaptation in response to inflammatory stimuli and the mechanistic basis for the long-term prothrombotic risk observed in patients with chronic or autoimmune inflammatory disease. Furthermore, the prothrombotic nature of trained myeloid cells warrants further consideration in relation to approaches to modulate trained immunity for therapeutic purposes (*27*, *64*–*68*).

## MATERIALS AND METHODS

### Generation of BMDMs

Female wild-type C57/BL6 mice aged between 6 and 12 weeks were euthanized by cervical dislocation, and hind legs were dissected, removed, and stored in high-glucose Dulbecco’s modified Eagle’s medium (DMEM; Merck) supplemented with 10% fetal bovine serum (FBS; Thermo Fisher Scientific), penicillin (1 U/ml), and streptomycin (0.1 mg/ml; Merck). Macrophages were isolated as previously described (*69*, *70*). Isolated macrophages were resuspended in supplemented DMEM containing 20% macrophage colony-stimulating factor (M-CSF) derived from L929 cells and incubated for 6 days, receiving 1 ml of M-CSF and 4 ml of fresh growth media on day 3. After 6 days, macrophages were detached using phosphate-buffered saline (PBS) containing 5 mM EDTA (Merck) and plated for experiments in complete DMEM containing 10% M-CSF.

### Isolation and culture of human monocytes

Anonymized healthy donor buffy coats were obtained from the Irish Blood Transfusion Service, St. James’ Hospital, Dublin, and monocytes were isolated as previously described (*71*). Briefly, PBMCs were isolated from buffy coats by layering blood over Histopaque-1077 gradient (Merck) and centrifuging at 400*g* for 25 min with acceleration and brake set to 1. Monocytes were then isolated using a Percoll (Merck) density centrifugation with 48.5% Percoll, 41.5% sterile water (Sigma-Aldrich), and 0.16 M NaCl (Merck) at 600*g* for 15 min with acceleration and brake set to 1. Monocytes were plated in serum-free RPMI containing 10 mM sodium pyruvate for 1 hour before changing media to complete RPMI supplemented with 10% human serum (from human male AB plasma, Merck), penicillin (1 U/ml), and streptomycin (0.1 mg/ml). Alternatively, a pure monocyte population was obtained from PBMCs using CD14+ Microbeads (Miltenyi Biotec).

### Innate immune cell training in BMDMs

BMDMs were plated at 5 × 10^5^ cells per well in 12-well plates for RNA isolation or 5 × 10^4^ cells/ml for TGA, plasmin generation assay (PGA), and enzyme-linked immunosorbent assays (ELISAs) in 96-well plates with complete DMEM and 10% M-CSF and left overnight to adhere. Cells were treated with either LPS (100 ng/ml; Enzo Life Sciences), β-glucan (100 μg/ml; Invivogen), 50 μM hemin (Merck), 50 μM protophorphyrin (Merck), or 50 μM Fe-NTA. Treated cells were left for 24 hours before aspirating media and washing three times with prewarmed PBS. DMEM containing 10% M-CSF was added to wells, and cells were left for a 7-day rest period. During this period, M-CSF and media were changed every 3 days. On day 7, all treatment wells (excluding control wells) were incubated with LPS (100 ng/ml) before being washed with PBS and either lysed for gene expression analysis or included in other assays.

### Innate immune cell training in human monocytes

Human monocytes were plated at 2 × 10^5^ cells per well in 96-well plates and cultured for 2 to 3 days in RPMI supplemented with 10% human serum (from human male AB plasma, Merck), penicillin (1 U/ml) and streptomycin (0.1 mg/ml). Cells were then treated with LPS (100 ng/ml; Enzo Life Sciences), β-glucan (100 μg/ml; Invivogen), or 10 to 50 μM hemin (Merck) for 24 hours. Following incubation, cells were gently washed three times with PBS, and growth media were replaced. Cells were left to rest for 5 days with media replaced on day 3. Cells were restimulated with LPS (100 ng/ml) on day 5. On the day of assay, cell plates were centrifuged at 1200 rpm for 5 min before removal of supernatants and gentle washing of cells with PBS. After this, downstream assays were performed.

### Myeloid cell–dependent TGA

Myeloid cell–dependent TGA was performed as previously described (*24*). Briefly, following cell stimulations, supernatants were removed, and cells were washed once with PBS. MP-reagent (20 μl) was added to 80 μl of FXII-deficient plasma (Protylix). To examine the procoagulant activity of each cell releasate, 80 μl of supernatants was added to plasma and MP reagent. The assay was initiated using 20 μl of FluCa-kit. Fluorescence was quantified using Thrombinoscope software on a Fluoroskan fluorometer. To block TF procoagulant activity, cells or supernatants were incubated with TF neutralizing antibody (10 μg/ml; 1H1, Genetech) for 1 hour before performing the TGA.

### Murine plasma TGA

The thrombin generation potential in murine plasma was determined without exogenous TF trigger. The assay was performed with wells containing 20 μl of murine plasma, 20 μl of tris-buffered saline (TBS), and 20 μl of FluCa-kit to initiate the reaction. The temperature for the assay run was lowered to 33°C on the fluorometer to overcome the increased coagulation inhibitor activity in mouse plasma compared to human plasma, and calibrator activity on Thrombinoscope software was set to 20% higher to account for a lower rate of conversion of the thrombin substrate by murine thrombin. These optimizations were based on the previous literature (*72*). Fluorescence was quantified using Thrombinoscope software on a Fluoroskan fluorometer.

### Myeloid cell–dependent PGA

Myeloid cell–dependent PGA was performed as previously described (*24*). Briefly, following cell stimulations, macrophages were washed with PBS and then incubated with 100 μl of serum-free DMEM supplemented with penicillin (1 U/ml), streptomycin (0.1 mg/ml), tissue plasminogen activator (tPA; 200 ng/ml), and 400 nM Glu-plasminogen (Protylix) for 1.5 hours. Supernatants were then transferred to a black flat-bottom 96-well plate. A Boc-Glu-Lys-Lys fluorometric substrate (20 μl) was added to each well to enable plasmin detection (final concentration, 1260 μM in TBS containing 34 mM CaCl_2_; Bachem). Assays were performed with each treatment group in duplicate per assay and at least biological triplicates, with a negative control well containing only serum-free DMEM. Fluorescence was quantified on a Fluoroskan Ascent fluorometer.

### FXa generation assay

Cell surface TF activity was measured as the ability of human monocyte monolayers to support FXa generation in the presence of activated FVIIa and CaCl_2_, as previously described (*73*, *74*). Monocytes were seeded at 1 × 10^6^ cells/ml in 96-well plates. Briefly, cells were washed twice with buffer A (10 nM Hepes, 0.15 M NaCl, 4 mM KCl, and 11 mM glucose, pH 7.5), after which cells were incubated with 100 μl of buffer B [buffer A containing bovine serum albumin (BSA; 5 mg/ml) and 5 mM CaCl_2_] containing FVIIa (0.5 μg/ml; Protylix) and FX (10 μg/ml; Protylix) for 30 min. After this time, 25 μl was collected from each well and added to 50 μl of buffer C [50 mM tris-HCl and 0.15 M NaCl, pH 7.5, with BSA (1 mg/ml) and 25 mM EDTA]. Fifty microliters of this mixture was then transferred to a new 96-well plate, and 50 μl of the substrate (1.25 mg/ml) was added to each well. The plate was then read at 405 nm for 1 hour, taking a reading every 30 s. The amount of FXa generated in the sample wells was calculated by interpolating from the *V*_max_ of an FXa standard curve starting at 1000 ng/ml using serial dilutions of purified human FXa (Protylix). To block TF activity, monocytes were incubated with TF neutralizing monoclonal antibody (4 μg/ml; HTF1, Thermo Fisher Scientific) for 1 hour before performing FXa generation assay.

### Myeloid cell–dependent prothrombinase activity assay

To determine prothrombinase activity as previously described (*75*), BMDM monolayers were incubated with FVa (10 nM) and FXa (1 nM) in 10 mM Hepes, 0.15 M NaCl, 4 mM KCl, and 11 mM glucose (pH 7.5) containing 5 mM CaCl_2_, 1 mM MgCl_2_, and BSA (1 mg/ml) for 5 min at 37°C. After this time, prothrombin (1.25 μM) was added to cells for a 1-min activation period. The reaction was then stopped by taking 50 μl of the above mixture and quenching with 50 μl of TBS containing 10 mM EDTA. The thrombin generated in each sample was then measured by adding 10 μl of chromogenic thrombin substrate [CS-01(81) Biophen; 1 mg/ml] to 40 μl of each sample. A kinetic reading at 405 nm was taken with a microplate reader, and the reading was converted to thrombin concentration using a calibration curve made with human α-thrombin.

### EV isolation and nanoparticle tracking analysis

To induce EV shedding, cells were serum starved in DMEM serum-free medium overnight at 37°C, after which conditioned media were harvested and centrifuged for 10 min at 1000*g* to remove cell debris. To pellet EVs, the supernatant was spun at 20,000*g* for 1 hour, as previously described (*76*). Nanoparticle tracking analysis was performed to measure the number and size of EVs released from BMDMs under different conditions. Particle size distribution in cellular supernatants was determined using a NanoSight NS300 system (Malvern Technologies) configured with a 488-nm laser and a high-sensitivity scientific complementary metal-oxide semiconductor camera. Samples were diluted to an acceptable concentration according to the manufacturer’s recommendations (1:20 dilution resulted in average particles per milliliter of 3 × 10^8^/ml). Three successive videos were captured per sample. Analysis was performed using NTA 3.1 software.

### Western blot analysis of isolated EVs

EVs were assessed via Western blotting as previously described (*77*). In short, EVs were lysed in 2× sample buffer (Life Technologies) and denatured for 5 min at 95°C, and equal volumes were loaded onto Bolt 4 to 12% Bis-Tris Plus WedgeWell Gels (Thermo Fisher Scientific), run for 40 min at 200 V, blotted on 0.2 μm–pore size polyvinylidene difluoride membranes, and blocked in 3% milk in TBS with 0.1% Tween 20 for 1 hour at room temperature. Blots were incubated with an anti-goat mouse TF (R&D Systems) or anti-rat mouse CD9 (Bio-Rad) antibody overnight at 4°C, washed three times with TBS with 0.1% Tween 20, and incubated with horseradish peroxidase secondary anti-goat (Jackson ImmunoResearch) or anti-rat antibody at room temperature for 1 hour. Peroxidase activity was visualized with Pierce ECL Western Blotting Substrate (Thermo Fisher Scientific) using the Amersham Imaging system (Cytiva). The band intensity was quantified via FIJI 2 software.

### mRNA isolation and RT-qPCR

A total of 5 × 10^5^ cells/ml was lysed using 350 μl of lysis buffer and total RNA isolated according to the manufacturer’s instructions (Ambion PureLink RNA isolation kit). Gene expression was determined by performing reverse transcription qPCR (RT-qPCR) in duplicate with Power Up SYBR green master mix (Thermo Fisher Scientific) using a 7500 Fast system (Applied Biosystems). Relative fold changes in mRNA expression were calculated using the cycle threshold (*C*_T_) and normalized to the *RPS18* housekeeping gene. Primer sequences are listed in table S1.

### ELISAs

PAI-1, TNFα, IL-6, and IL-1β levels released by treated macrophages were determined using DuoSet ELISA kits (R&D Systems) per the manufacturer’s protocol. Murine thrombin-antithrombin levels were determined in plasma and BM interstitial fluid samples using an ELISA kit (Abcam).

### Macrophage siRNA transfection

SMARTpool siRNA guides were designed by Horizon Discovery and chosen using the siGENOME siRNA search tool. siRNAs (20 nM) were transfected using Lipofectamine RNAiMax (Invitrogen). Cells were seeded at 2.5 × 10^5^ cells per well on 24-well plates. On the day of transfection, media were removed and replaced with 400 μl of OptiMEM (Gibco) containing 5% M-CSF. siRNAs were transfected into cells at a concentration of 20 nM using Lipofectamine RNAiMax (Invitrogen). Untransfected controls containing OptiMEM were also included in each experiment. The following day, media were removed and replaced with DMEM containing 10% M-CSF derived from L-929 cells. Twenty-four hours following transfection, cells were treated as required for downstream assays.

### In vivo trained immunity model

Male C57BL/6J-OlaHsd mice aged 8 to 12 weeks were used. All experiments were performed under the approval of the Health Products Regulatory Authority, Ireland and Trinity College Dublin Animal Research Ethics Committee (AE19136/P148). Trained immunity was induced in mice with a single intraperitoneal injection of either 200 μl of PBS as a control or 200 μl of whole glucan particles (Invivogen) resuspended in PBS at 1 mg/ml. At week 1, 2, 3, or 4 postinjection, whole blood was collected from animals via the retro-orbital sinus into 3.2% sodium citrate-coated tubes and plasma was obtained by centrifuging at 2000 rpm at 4°C for 15 min. Mice were euthanized by CO_2_ inhalation, and spleens and legs were harvested. To assess the effect of dietary β-glucan, mice were fed a diet supplemented with dietary fiber whole glucan particle (Kerry Food group). After 4 weeks, mice were euthanized by CO_2_ inhalation and spleens were harvested as before.

### Isolation of splenic monocytes and macrophages

Spleens were homogenized and passed through a 70-μm cell strainer (BD) with RPMI 1640 media supplemented with 10% FBS, penicillin (1 U/ml), and streptomycin (0.1 mg/ml) to obtain a single-cell suspension for each sample. Each cell suspension was centrifuged at 1200 rpm for 5 min, and the supernatant was discarded. Splenocytes were then resuspended in 1 ml of red cell lysis buffer for 2 min before washing with 10 ml of complete RPMI media. Each sample was centrifuged again at 1200 rpm for 5 min, the supernatant was discarded, and cells were resuspended in 5 ml of complete RPMI. Monocytes were then isolated using anti-CD115 microbeads (Miltenyi Biotec) according to the manufacturer’s protocol. Following isolation, cells were centrifuged and resuspended in RPMI supplemented with 10% FBS, penicillin (1 U/ml), and streptomycin (0.1 mg/ml) before plating at the required cell densities. Cells were plated at 5 × 10^4^ cells per well on 96-well plates for functional assays. Cells were left to adhere overnight, and fresh growth media were added the following day. Cells were then treated with LPS (100 ng/ml) for 24 hours for functional assays.

### Collection of BM interstitial fluid

To collect BM interstitial fluid, mouse femurs were flushed with 500 μl of cold PBS, and the fluid was centrifuged at 500*g* for 5 min at 4°C. The BM fluid was then collected, transferred to new Eppendorf tubes, and stored at −80°C for further analysis.

### Cell Mito Stress Test

Oxygen consumption rate and extracellular acidification rate were analyzed in BMDMs using the Seahorse XFe96 Analyzer (Agilent). The Cell Mito Stress Test assay was performed according to the manufacturer’s instructions. A total of 5 × 10^4^ cells per well was seeded onto a Seahorse XF96 cell culture microplate and left to adhere overnight. BMDMs were then trained as described. BMDMs were then restimulated with LPS (100 ng/ml) for 6 hours before performing a Cell Mito Stress Test. Supernatants were removed and replaced with assay medium [Seahorse XF DMEM (Agilent) supplemented with 1 mM pyruvate, 2 mM glutamine, and 10 mM glucose (Merck)]. The assay was performed with sequentially injected 1 μM oligomycin, 1 μM carbonyl cyanide p-trifluoromethoxyphenylhydrazone, and 0.5 μM rotenone/antimycin A. All measurements were performed with eight technical replicates per experiment. Once the assay was completed, data were normalized to total protein per well determined by a BCA Protein Assay Kit (Thermo Fisher Scientific). Alterations in cellular metabolism were assessed by changes in oxygen consumption rate and extracellular acidification rate using Wave Desktop software.

### Flow cytometry

Following each treatment, macrophages were incubated in PBS containing 5 mM EDTA for 5 min to remove cells from 12-well plates. Cells were washed with 1 ml of PBS and stained with Live/Dead dye (Invitrogen) for 30 min at 4°C in the dark. Cells were left on ice for 30 min in the dark, after which FACS tubes were centrifuged at 1200 rpm for 5 min and supernatants were decanted. Cells were then incubated with an anti-CD16/CD32 monoclonal antibody to block FC receptors (Invitrogen). Cells were stained with murine anti-F4/80 antigen PE-conjugated antibody (Invitrogen), murine anti-TF 1H1 antibody (Genentech), and ASMase Polyclonal antibody (Invitrogen) on ice in the dark for 1 hour The secondary antibody for TF staining was goat anti-rat IgG Alexa Fluoro 568 (Invitrogen), while the secondary for ASMase staining was goat anti-rabbit IgG Alexa Fluoro 488 (Invitrogen). Cells were washed and resuspended in 300 μl of FACS Buffer and analyzed using the BD FACS Canto flow cytometer for TF expression and annexin V and the Attune NxT flow cytometer for F4/80 and ASMase expression.

### Measurement of PS exposure on BMDMs

PS expression was assessed using annexin V and propidium iodide (PI) staining. For annexin V staining, all washes, incubations, and measurements were performed in annexin V binding buffer (Thermo Fisher Scientific). PI staining was performed in PBS. BMDMs were incubated with annexin V-FITC (1 μg/ml; Thermo Fisher Scientific). After 5 min, cells were washed and resuspended in 200 μl of binding buffer. PI was added directly to FACS tubes (1 μg/ml) immediately before acquiring the samples on the BD FACS Canto flow cytometer.

### Measurement of ASMase activity

Trained macrophages were restimulated with LPS (100 ng/ml) on day 7 for 30 min, 1 hour, or 3 hours before cells were lysed. ASMase activity in the cell lysates was then measured using an ASMase activity assay kit (Echelon Biosciences) according to the manufacturer’s instructions.

### Flow cytometry analysis of HSPC populations

To analyze HSPC populations in mouse BM after in vivo induction of trained immunity, isolated BM cells were isolated and cKit^+^ cells were enriched for using cKit Microbeads (Miltenyi). Isolated cKit^+^ HSPCs were resuspended in flow buffer [1× PBS (Gibco) supplemented with 2% heat-inactivated FBS (Gibco)]. A flow cytometry staining protocol was applied to all BM samples, as previously described (*18*). Cells were stained with fixable viability stain ZombieAqua (BioLegend) at 1:1000 for 15 min. The following antibodies were then used for staining LSK cells, HSCs, and MPPs: Anti-Ter-119 (clone TER-119, BioLegend), anti-CD5 (clone 53-7.3, BioLegend), anti-CD8a (clone 53-6.7, BioLegend), anti-B220+ (clone RA3-6B3, BioLegend), and anti-Ly6G/C+ (clone RB6-8C5, BioLegend), all conjugated to APC-Cy7, were added at 1:200 for 25 min. Other extracellular antibodies for anti-c-Kit-APC (clone 2B8, BioLegend), anti-Sca-1-PE-Cy7 (clone D7, eBioscience), anti-CD150-PE (clone TC15-12F, eBioscience), anti-CD48-PerCP-eFluor710 (clone HM48-1, BD Bioscience), anti-CD34-FITC (clone RAM34, eBioscience), and anti-Flt3-BV421 (clone A2F10.1, BioLegend) were added at 1:50 and incubated at 4°C for 25 min. All cells were washed with flow buffer and resuspended with IC Fixation Buffer (Invitrogen). Compensation controls were obtained after staining UltraComp eBeads Compensation Beads (Invitrogen) with the appropriate antibodies. Absolute counts were obtained using Precision Count Beads (BioLegend). Cells were acquired on the BD flow cytometer Fortessa with FACSDiva software. Data analysis was performed using FlowJo software. See fig. S9 for the gating strategy used.

### RNA sequencing and analysis

Splenic monocytes were isolated from control and β-glucan–injected mice and restimulated ex vivo with LPS (100 ng/ml) for 6 hours. Supernatants were removed, cells were washed once with PBS, and cells were lysed with lysis buffer (Ambion PureLink RNA isolation kit) containing 1% 2-mercaptoethanol. Total RNA was isolated according to the manufacturer’s instructions. RNA samples were purified using RNeasy PowerClean Pro Cleanup Kit (Qiagen). Sample quality control and RNA library preparation were performed in-house by Novogene, Cambridge. RNA sequencing was performed using Illumina PE150 technology. Raw data from RNA sequencing were then bioinformatically processed in-house by Novogene. Briefly, this included quality control of reads where reads were removed if they included adaptor contamination, >10% of bases were resolved as uncertain (N), and >50% of bases had a base quality <5. All samples had >97% of bases with Q Phred values greater than 20 and more than 93% of bases with a Q Phred value greater than 30. With these filtered samples and reads, reads were aligned to the mouse GRCm38 reference using HISAT2 (*78*). Using the counts of reads to each mapped gene as input, differential gene expression analysis was carried out with the R package DESeq2 (*79*) using the recommended pipeline by its authors. The enrichment analysis was carried out using clusterProfiler software (*80*).

Volcano plot visualization of log_2_ fold change and log_10_-transformed *P* values, Gene Ontology enrichment visualization, and heatmap visualization of gene expression profiles of LPS-restimulated β-glucan–trained BMDMs compared to LPS-treated BMDMs were carried out using the statistical computing language R version 4.3.2 (*81*) and the ggplot2 R package (*82*).

### Statistical analysis

Data were analyzed for normal distribution (Shapiro-Wilk or Kolmogorov-Smirnov test) and equality of variance (*F* test). One-way analysis of variance (ANOVA) analysis was used to compare the means of three or more groups. Where significance was found in the ANOVA analysis, Tukey’s multiple comparison test was used to define differences between individual groups. To compare the mean of the two groups, Student’s *t* test was used, unpaired for in vivo experiment analysis and paired for in vitro experiment analysis or Mann-Whitney *U* test for nonparametric data, as appropriate. All analyses and graph representations were performed using GraphPad Prism 9 software, and data are shown as means ± SD.
