# Supplementary material for: Trained immunity causes myeloid cell hypercoagulability
Source: Sci Adv. 2025 Mar 7;11(10):eads0105. doi: 10.1126/sciadv.ads0105 (PMC11887800; doi:10.1126/sciadv.ads0105)
Supplement: Supplementary file 1 — Figs. S1 to S12 Tables S1 to S3 [file sciadv.ads0105_sm.pdf]

Science Advances
AAAS

Supplementary Materials for

Trained immunity causes myeloid cell hypercoagulability

Aisling M. Rehill *et al.*

Corresponding author: Roger J. S. Preston, rogerpreston@rcsi.ie

Sci. Adv. **11**, eads0105 (2025)
DOI: 10.1126/sciadv.ads0105

This PDF file includes:

Figs. S1 to S12
Tables S1 to S3

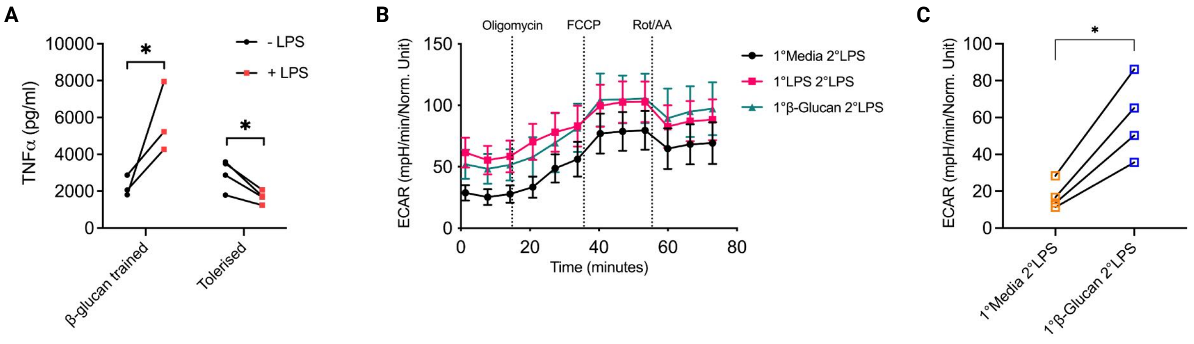

Fig. S1. β-glucan trained BMDMs have increased proinflammatory cytokine production and increased glycolysis.

BMDMs were pre-treated with media or 100 µg/mL whole glucan particle, left for 24 hr before cells were washed 3 times with PBS and left to rest for 1 week. On day 7, cells were restimulated with 100 ng/mL LPS. **(A)** TNFα production from BMDMs was measured by ELISA. **(B-C)** XF Seahorse Mito Stress Test was performed on β-glucan trained cells to determine extracellular acidification rate (ECAR) following the sequential addition of oligomycin, FCCP and Rotenone/antimycin. Results are shown as ECAR basal glycolysis. A paired t-test was used to determine statistical significance with *P≤0.05 for 3-4 independent experiments measured in duplicate.

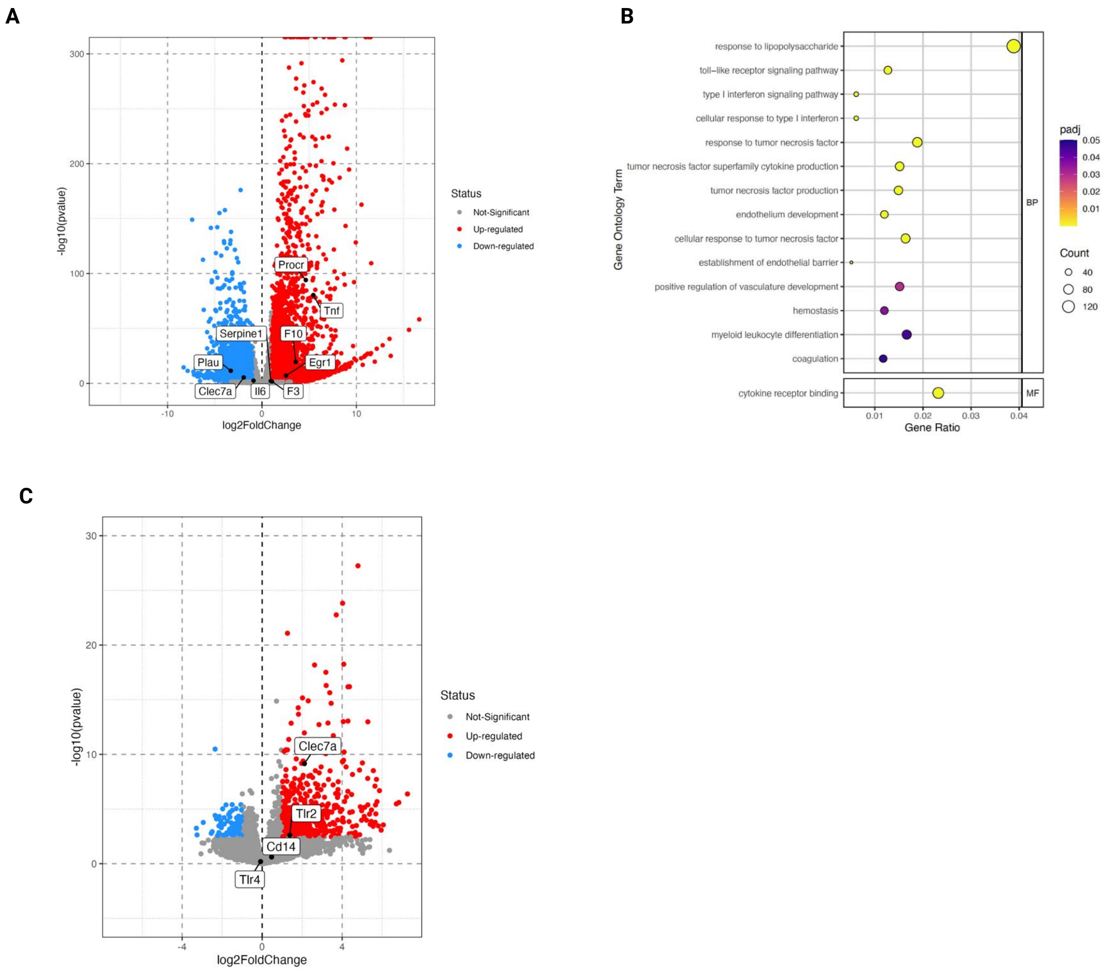

Fig. S2. RNAseq analysis of LPS-treated BMDMs and receptor expression in β-glucan-trained BMDMs. RNA sequencing was performed on β-glucan-trained (n=3) LPS-treated (n=3) and untreated BMDMs (n=3). (A) Differential expression of genes (DEGs) analysis carried out between LPS-treated and untreated BMDMs. **(B)** Gene Ontology (GO) enrichment analysis of the gene sets significantly upregulated in LPS-treated BMDMs compared to untreated BMDMs. (BP=biological processes; MF= molecular functions). **(C)** DEG analysis performed between β-glucan-trained and LPS-treated macrophages showing no significant change in LPS receptor expression.

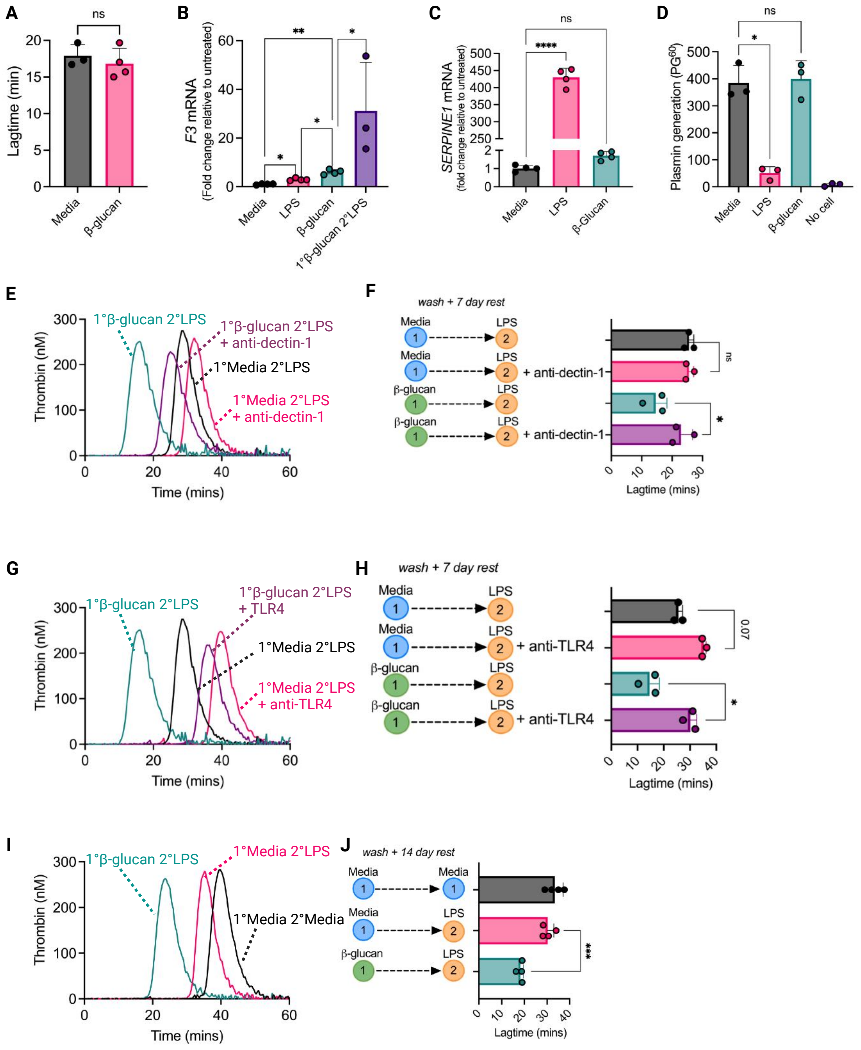

Fig. S3. Single treatment with β-glucan does not induce TF or PAI-1 activity BMDMs were treated with media or β-glucan for 24 hours before performing a TGA and RNA isolation. **(A)** TGA was performed with these BMDMs to generate lag-times. **(B)** *F3* and **(C)** *Serpine1* expression were determined by RT-qPCR. **(D)** t-PA-mediated plasmin generation in the presence of BMDMs was measured using a plasmin-specific fluorogenic substrate, and the fluorescence reading after 60 mins (PG^{60}) was determined. **(E)** BMDMs were incubated with anti-dectin-1 monoclonal antibody for 1 hr prior to β-glucan treatment. TGA was then performed on day 7 following LPS restimulation to determine **(F)** lagtimes. **(G)** BMDMs were incubated with 5 µg/mL anti-TLR4/MD2 monoclonal antibody for 1 hr prior to LPS restimulation. A TGA was then performed to determine **(H)** lagtimes. **(I)** BMDMs were trained with β-glucan and left for a 14 day rest period after which cells were restimulated with LPS and a TGA performed to determine **(J)** lagtimes. A paired t-test or one-way ANOVA was used where appropriate to determine statistical significance with $^*P \leq 0.05$, $^{**}P \leq 0.01$, $^{****}P \leq 0.0001$ for 3-4 independent experiments measured in duplicate.

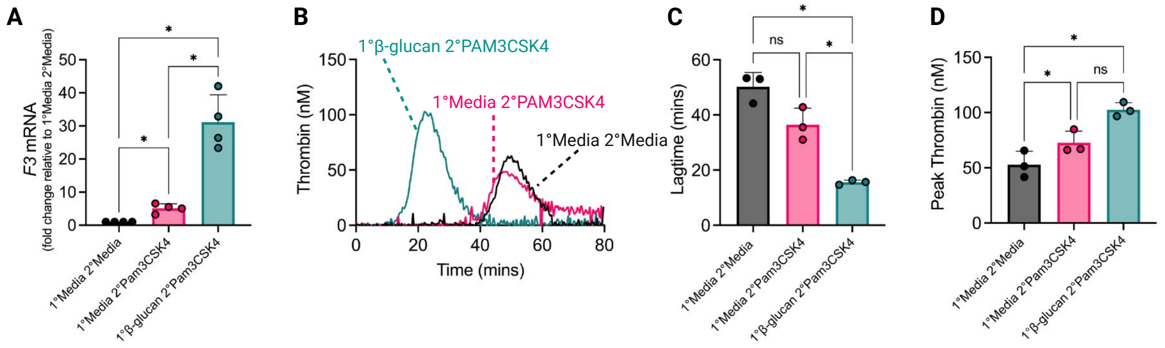

Fig. S4: PAM3CSK4 restimulation following β-glucan-mediated training also increases BMDM hypercoagulability. BMDMs were pre-treated with media or 100 µg/mL β-glucan, left for 24 hr before cells were washed 3 times with PBS and left to rest for 1 week. On day 7, cells were restimulated with 50 µg/mL PAM3CSK4. **(A)** *F3* mRNA levels were determined by RT-qPCR. **(B)** TGA was performed with PAM3CSK4 restimulated β-glucan primed-BMDMs and **(D)** associated lag-time and **(D)** peak thrombin determined. A paired t-test or one-way ANOVA was used where appropriate to determine statistical significance with $^{*}P \leq 0.05$, $^{**}P \leq 0.01$, $^{****}P \leq 0.0001$ for 3-4 independent experiments measured in duplicate.

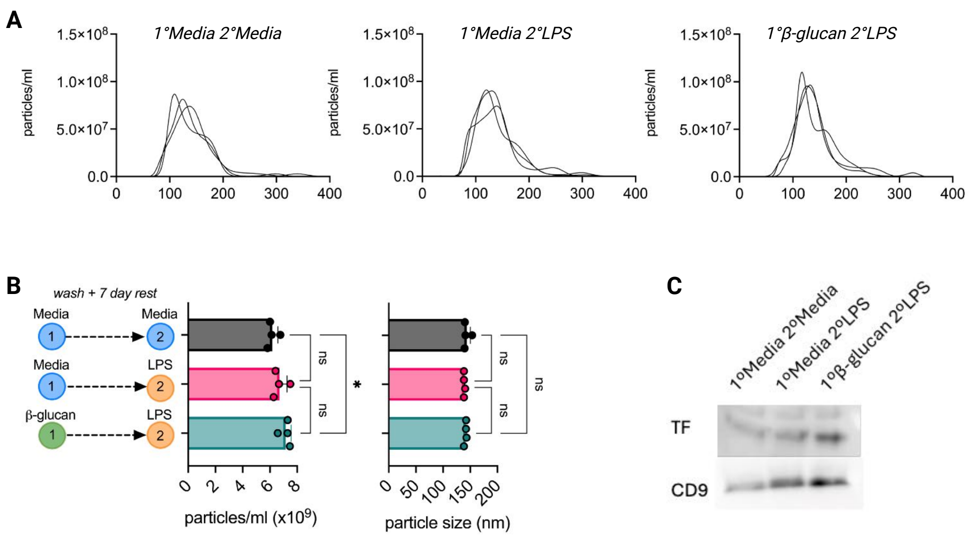

Fig. S5: Increased EV shedding in LPS-treated and β-glucan-trained BMDMs. The EV-enriched fraction was isolated from the supernatants of BMDMs treated with LPS and β-glucan. Particle size analysis using NTA was performed for 3 biological replicates. **(A)** Representative NTA size distribution traces from one replicate are displayed. **(B)** EV particle concentrations and particle size were averaged for each treatment, with histograms representing the average of three experiments. **(C)** CD9 and TF protein expression by isolated EVs was determined by western blot. A one-way ANOVA was used to determine statistical significance with $*P \leq 0.05$ for 3 independent experiments.

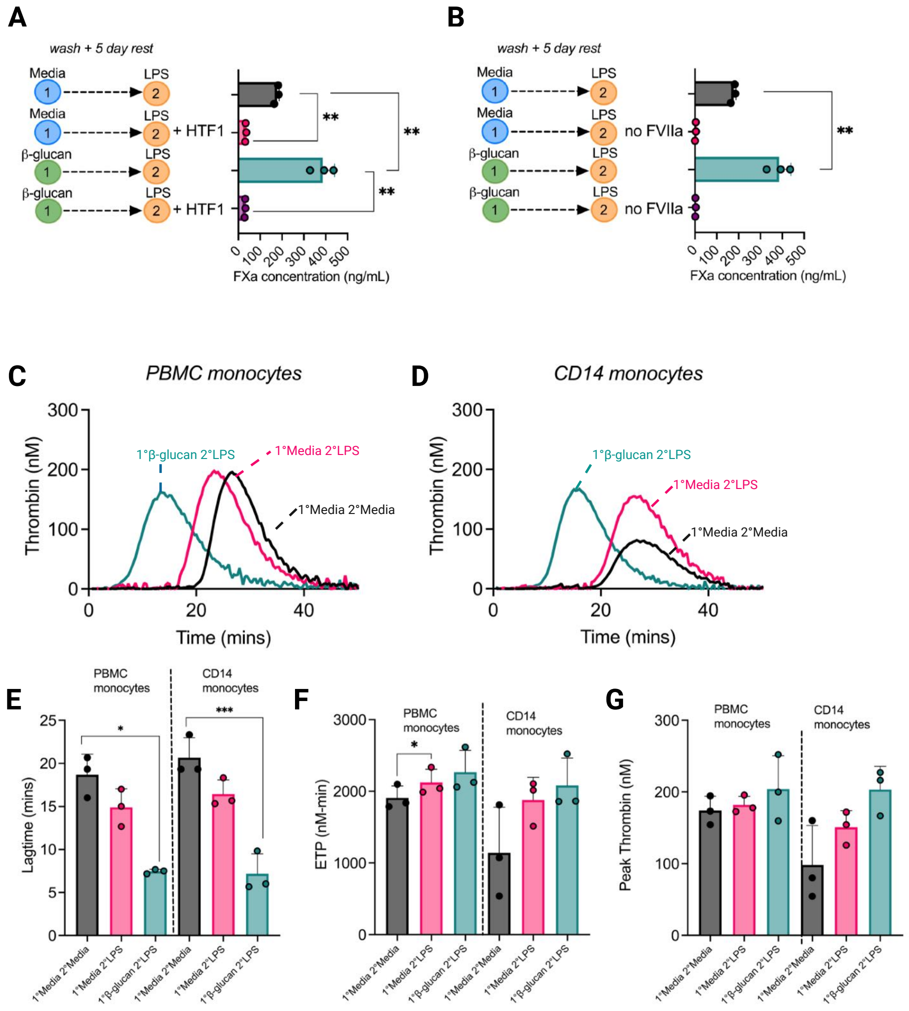

Fig. S6: IFNγ-producing lymphocytes are not required for enhanced procoagulant activity in β-glucan-trained monocytes. Peripheral blood mononuclear cells (PBMCs) were isolated from healthy donor buffy coats by gradient centrifugation using histopaque-1077. PBMCs were then plated in presence of sodium pyruvate to encourage adherence. Alternatively, purified monocytes were isolated from PBMC using $CD14^+$ positive selection microbeads. PBMC and CD14+ monocyte populations were primed with 100 µg/mL β-glucan, left for 24 hr before cells were washed 3 times with PBS and left to rest for 5 days. On day 5, cells were restimulated with 100 ng/mL LPS for 24 hr before FXa generation assay or TGA analysis was performed. **(A)** Monocytes were incubated with 4 µg/mL TF monoclonal antibody (HTF1) for 1 hr prior to performing FXa generation assay. **(B)** FXa generation was performed with monocytes in the presence or absence of FVIIa. Representative TGA thrombograms for **(C)** PBMC monocytes and **(D)** $CD14^+$ purified monocytes and associated parameters **(E)** lag-times, **(F)** ETP and **(G)** peak thrombin were determined. A one-way ANOVA was used to determine statistical significance with $^*P \leq 0.05$, $^{**}P \leq 0.01$ and $^{***}P \leq 0.001$ for 3-4 independent experiments measured in duplicate.

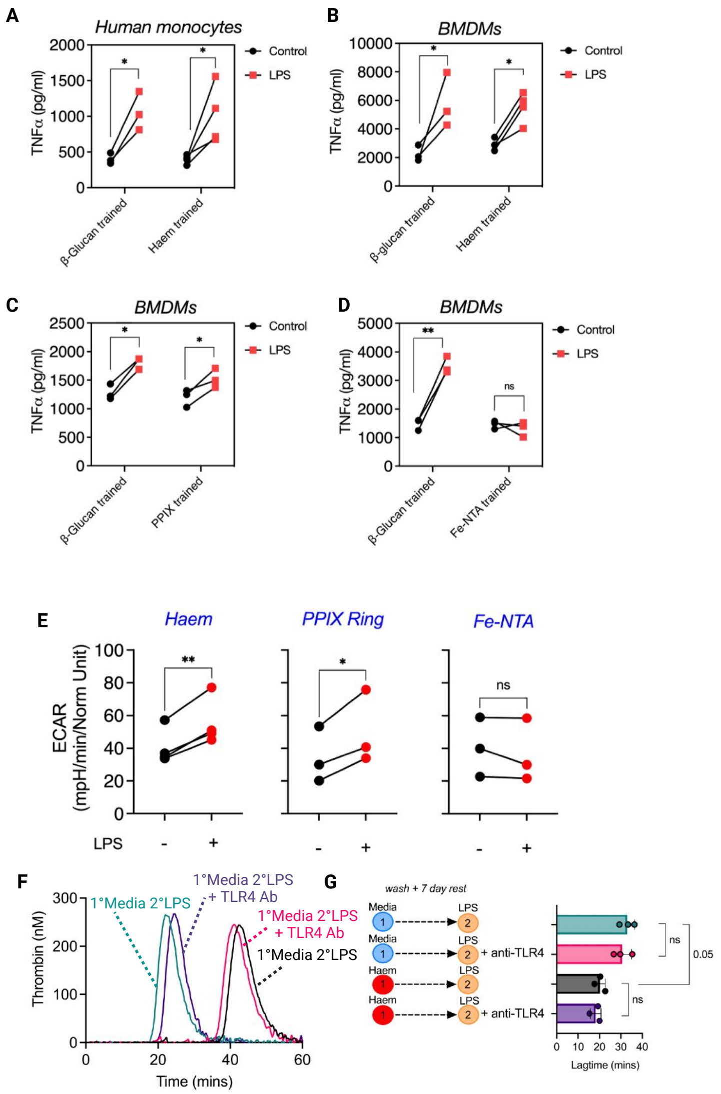

Fig. S7: Free haem induces trained immunity in myeloid cells through enhanced pro-inflammatory cytokine production and increased glycolysis. BMDMs and human monocytes were trained with 100 µg/mL β-glucan, 50 µM haem, 50 µM protoporphyrin IX (PPIX), 50 µM Fe-NTA or DMSO vehicle control. After 24 hrs, cells were washed and rested for 7 days. Cells were then restimulated with 100 ng/mL LPS. TNFα levels were measured by ELISA in **(A)** haem-trained human monocytes, **(B)** haem-trained BMDMs, **(C)** PPIX-trained BMDMs and **(D)** Fe-NTA-trained BMDMs. **(E)** XF Seahorse Mito Stress Test was performed on free haem-, PPIX- and Fe-NTA-trained BMDMs to determine ECAR following sequential addition of oligomycin, FCCP and Rotenone/antimycin. **(F)** BMDMs were incubated with 5 µg/mL TLR4/MD-2 complex monoclonal antibody for 1 hr prior to haem training. TGA performed lowing LPS restimulation on day 7 to determine thrombogram and **(G)** lagtime. A paired t-test was used to determine statistical significance with *P≤0.05 and **P≤0.01 for 3-4 independent experiments measured in duplicate.

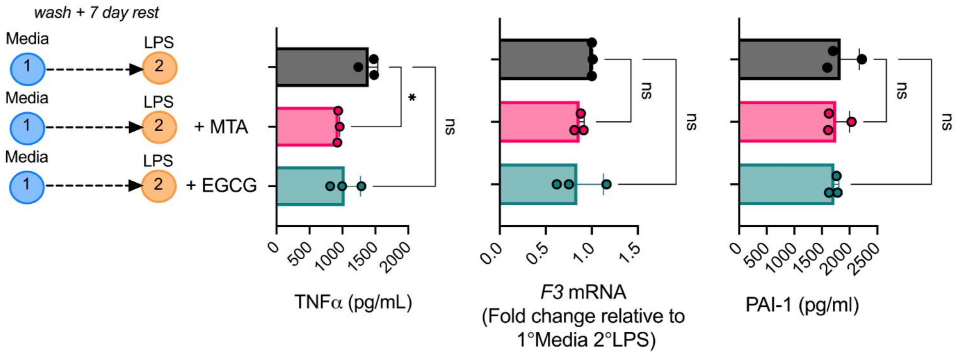

Fig. S8: Epigenetic inhibitors have limited effect on LPS-treated BMDMs. BMDMs were incubated with 1 mM MTA, 100µM EGCG or 12mM PG for 24 hours on day 1. Cells were washed 3 times and growth media supplemented with epigenetic inhibitors added for the rest period (100 µM MTA, 100 µM EGCG and 12 mM PG). On day 7, cells were stimulated with 100 ng/mL LPS. *F3* mRNA levels in β-glucan-trained BMDMs were determined by RT-qPCR, and TNFα and PAI-1 protein levels were determined by ELISA.

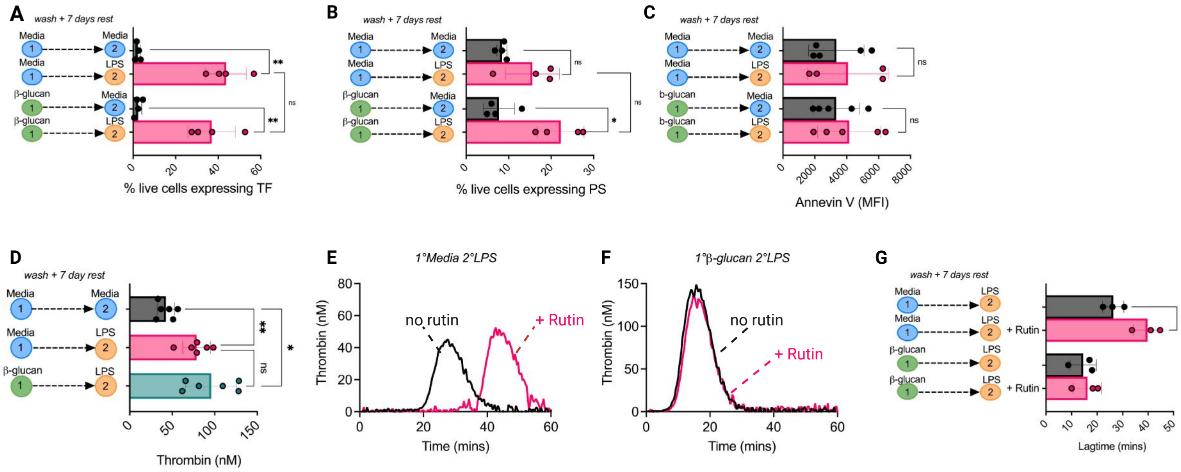

Fig. S9: β-glucan-mediated trained immunity does not increase TF antigen expression, PS externalisation or protein disulfide isomerase (PDI). BMDMs were pre-treated with media or 100 µg/mL β-glucan, left for 24 hr before cells were washed 3 times with PBS and left to rest for 1 week. On day 7, cells were either left untreated or restimulated with 100 ng/mL LPS. **(A)** TF surface expression on live cells was measured by flow cytometry. Phosphatidylserine exposure was measured by examining Annexin V binding by flow cytometry with results shown as **(B)** % cells expressing PS and **(C)** mean fluorescence intensity (MFI). **(D)** A prothrombinase assay was performed in presence of BMDMs by incubating cells with 10 nM FVa and 1nM FXa for 5 mins after which time 1.25 µM prothrombin was added for 1 min. The amount of thrombin generated was determined by adding a thrombin chromogenic substrate. Rutin, a potent inhibitor of PDI, was incubated (100 µM) with β-glucan and LPS-treated BMDMs for 1 h before TGA analysis. Representative thrombograms for **(E)** LPS-treated BMDMs, **(F)** β-glucan-trained BMDMs and **(G)** associated lag-times were determined. A paired t-test was used to determine statistical significance with *P≤0.05 and **P≤0.01 for 3-4 independent experiments measured in duplicate.

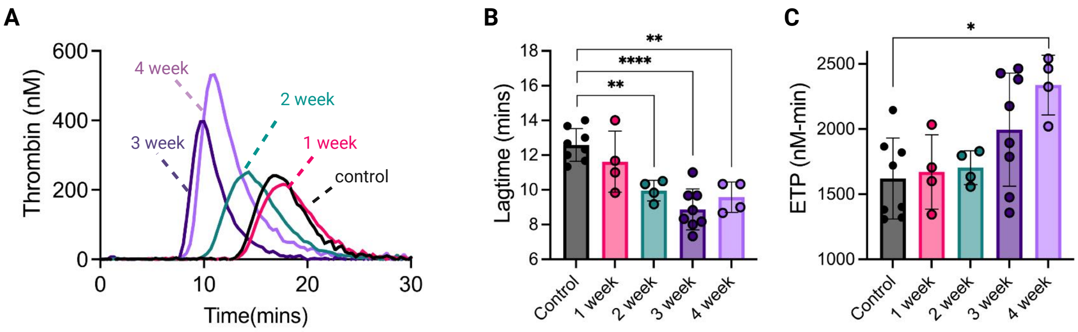

Fig. S10: *Ex vivo* LPS stimulation of splenic monocytes from β-glucan administered mice is not required for enhanced procoagulant activity. β-glucan mice were injected with either PBS or whole glucan particles 1-4 weeks before sacrifice. $CD115^+$ splenic monocyte population were then isolated. Splenic monocytes were restimulated 100 ng/mL LPS *ex vivo* for 24 hr. A TGA was performed in the presence of LPS-treated splenic monocytes to generate **(A)** thrombogram, **(B)** lag-time and **(C)** ETP values. A One-Way ANOVA was used to determine statistical significance with *P≤0.05, **P≤0.01 and ****P≤0.0001 for 4-8 mice with samples measured in duplicate.

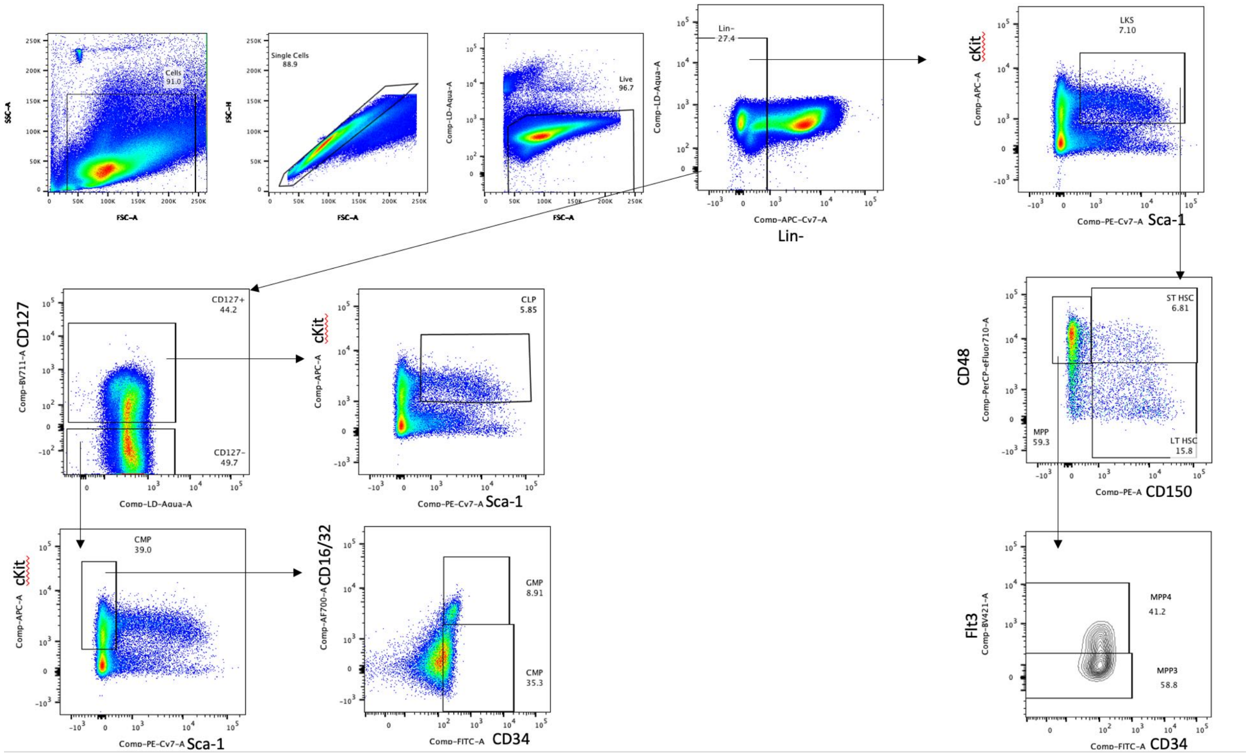

Fig. S11: Gating strategy for flow cytometry analysis of haematopoietic stem and progenitor cell (HSPCs) populations. Flow cytometry gating strategy for haematopoietic stem cells (HSCs) and multipotent progenitor (MPP) populations. LSK ($Lin^{-}cKit^{+}Sca1^{+}$), Common myeloid progenitor (CMP; $Lin^{-}CD127^{-}ckit^{+}Sca\text{-}1^{-}CD34^{+}CD16/32^{-}$) granulocyte macrophage progenitors (GMP; $Lin^{-}CD127^{-}ckit^{+}Sca\text{-}1^{-}CD34^{+}CD16/32^{+}$), common lymphoid progenitors (CLP; $Lin^{-}CD127^{+}Ckit^{+}Sca1^{+}$), myeloid biased MPP3 ($Sca\text{-}1^{+}ckit^{+}CD48^{+}CD150^{-}Flt3^{-}$ and lymphoid biased MPP4 ($Sca\text{-}1^{+}ckit^{+}CD48^{+}CD150^{-}Flt3^{+}$).

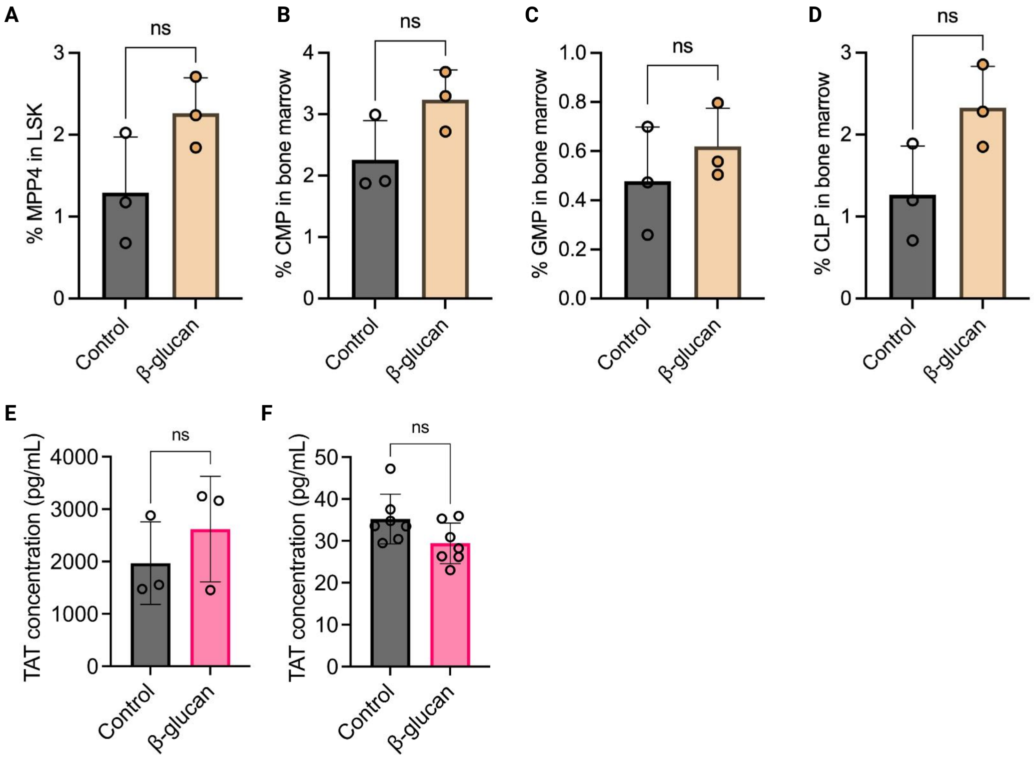

Fig. S12: No significant change in lymphoid progenitor populations or TAT levels in β-glucan administered mice. Mice were injected with either PBS or whole glucan particles 1-4 weeks prior to sacrifice. BM haematopoietic stem and progenitor cell populations were analysed by flow cytometry for **(A)** % MPP4 lymphoid-biased progenitor cells in the LSK^+ compartment, and **(B)** % CMP, **(C)** % GMP, **(D)** % CLP in the bone marrow. TAT levels were determined by ELISA in **(E)** plasma and **(F)** bone marrow interstitial fluid samples. An unpaired t-test was used to determine statistical significance for 3 biological replicates.

Primer		Sense			Antisense	
**MURINE GENES**						
RPS18	5’	CTTAGAGGGACAAGTGGCG	3’	5’	ACGCTGAGCCAGTCAGTGTA	3’
PROCR	5’	CAGGACACTTGTGTGGAGTT	3’	5’	CCAGGACCAGTGATGTGTAAG	3’
F10	5’	GAAGAGCAGAACTCAGTGGTGTG	3’	5’	CAAGCTCACTGTCGCTGGTGTT	3’
F3	5’	GCACCGAGCAATGGAAGAGTTTC	3’	5’	CTTTCTGTCCCGCTCGGTTCTT	3’
SERPINC1	5’	TCCGACGCATTCCACAAA	3’	5’	GGCCAGTAATCACGACAGAA	3’
F7	5’	CAGCCCACTGCTTCGATAATA	3’	5’	GTGTCACCCGTCGTACTTG	3’
F11	5’	GATTACCAAGCACACGCATTAC	3’	5’	AAGGACGGATGGCAGAATAC	3’
F9	5’	GCATTCTGTGGAGGTGCCATCA	3’	5’	TCTCCTTTGTTCTGTGTCTTCCTT	3’
THBD	5’	GGAGAATGGTGGCTGTGAGTAC	3’	5’	GCACGATTGAACCACAGGTCTTG	3’
TFPI	5’	AGGGAACGAGAACCGATTTG	3’	5’	TGCCTTCACAGCTGTCTTC	3’
PROS1	5’	TGGCAAGGAGACAGGTGTCAGT	3’	5’	GAGCAGTGGTAACTTCCAGGAG	3’
F2	5’	ACCTTGGGACTGTGAATGTC	3’	5’	GATGGGTGGTGGAGTTGATT	3’
F8	5’	GGCGAGTAGAATGCCTTATTGGC	3’	5’	ATCACGGATGCTTCCAGAAGCC	3’
F5	5’	TGATGCTGTCCAGCCCAATAGC	3’	5’	CGATCAAGCCTGAGTGGATGTC	3’
SERPINE1	5’	CCTCTTCCACAAGTCTGATGGC	3’	5’	GCAGTTCCACAACGTCATACTCG	3’
EGR1	5’	AGCGAACAACCCTATGAGCACC	3’	5’	ATGGGAGGCAACCGAGTCGTTT	3’

Table S1. RT-qPCR primer sequences for murine and human genes

gene_id	log2FoldChange	pvalue	padj	gene_name	status
ENSMUSG00000021822	3.1286	2.21e-11	8.98e-9	*Plau*	Up-regulated
ENSMUSG00000079293	2.1111	7.04e-10	1.82e-7	*Clec7a*	Up-regulated
ENSMUSG00000027834	2.4216	8.17e-9	1.73e-6	*Serpine1*	Up-regulated
ENSMUSG00000022126	1.1916	2.48e-9	5.8e-7	*Acod1*	Up-regulated
ENSMUSG00000038418	1.2857	4.32e-7	4.84e-5	*Egr1*	Up-regulated
ENSMUSG00000024401	1.1995	5.96e-7	6.42e-5	*Tnf*	Up-regulated
ENSMUSG00000025746	1.8198	1.69e-4	0.00563	*Il6*	Up-regulated
ENSMUSG00000028128	2.9656	4.12e-5	0.00195	*F3*	Up-regulated
ENSMUSG00000027611	1.3692	0.00254	0.04093	*Procr*	Up-regulated
ENSMUSG00000031444	1.7185	0.00473	0.06114	*F10*	Not-Significant

Table S2. RNAseq Differential expression of genes (DEG) analysis results for β-glucan trained BMDMs (1°β-glucan 2°LPS) compared to LPS-treated BMDMs (1°Media 2°LPS)

gene_id	log2FoldChange	pvalue	padj	gene_name	status
ENSMUSG00000027611	4.64034442	8.43E-95	4.70E-93	*Procr*	Up-regulated
ENSMUSG00000024401	5.4380995	4.03E-81	1.75E-79	*Tnf*	Up-regulated
ENSMUSG00000031444	3.57005048	2.71E-20	2.34E-19	*F10*	Up-regulated
ENSMUSG00000038418	2.5470425	8.30E-08	3.15E-07	*Egr1*	Up-regulated
ENSMUSG00000027834	0.93246693	0.00838	0.01643	*Serpine1*	Up-regulated
ENSMUSG00000028128	1.05307692	0.02081	0.03768	*F3*	Up-regulated
ENSMUSG00000025746	-0.9012695	0.00349	0.00733	*Il6*	Down-regulated
ENSMUSG00000079293	-1.9342021	4.05E-06	1.28E-05	*Clec7a*	Down-regulated
ENSMUSG00000021822	-3.3089008	3.55E-12	1.91E-11	*Plau*	Down-regulated

Table S3. RNAseq Differential expression of genes (DEG) analysis results for LPS-treated BMDMs (1°Media 2°LPS) compared to untreated BMDMs (1°Media 2°Media)
